# RIG-I/MAVS and STING signaling promote gut integrity during irradiation- and immune-mediated tissue injury

**DOI:** 10.1126/scitranslmed.aag2513

**Published:** 2017-04-19

**Authors:** Julius C. Fischer, Michael Bscheider, Gabriel Eisenkolb, Chia-Ching Lin, Alexander Wintges, Vera Otten, Caroline A. Lindemans, Simon Heidegger, Martina Rudelius, Sébastien Monette, Kori A. Porosnicu Rodriguez, Marco Calafiore, Sophie Liebermann, Chen Liu, Stefan Lienenklaus, Siegfried Weiss, Ulrich Kalinke, Jürgen Ruland, Christian Peschel, Yusuke Shono, Melissa Docampo, Enrico Velardi, Robert R. Jenq, Alan M. Hanash, Jarrod A. Dudakov, Tobias Haas, Marcel R. M. van den Brink, Hendrik Poeck

**Affiliations:** 1 III. Medizinische Klinik, Klinikum rechts der Isar, Technische Universität München, Munich, Germany; 2 Department of Immunology, Memorial Sloan Kettering Cancer Center, New York, NY 10065, USA; 3 Pediatric Blood and Bone Marrow Transplant Program, University Medical Center Utrecht, Utrecht, Netherlands; 4 Department of Medicine, Memorial Sloan Kettering Cancer Center, New York, NY 10065, USA; 5 Institute of Pathology, University of Wuerzburg and Comprehensive Cancer Center Mainfranken, Wuerzburg, Germany; 6 Tri-Institutional Laboratory of Comparative Pathology, Memorial Sloan Kettering Cancer Center, Rockefeller University, and Weill Cornell Medical College, New York, NY 10065, USA; 7 Department of Pathology and Laboratory Medicine, New Jersey Medical School and Robert Wood Johnson Medical School, Rutgers University, Newark, NJ 08903, USA; 8 Institute for Experimental Infection Research, TWINCORE, Centre for Experimental and Clinical Infection Research, a joint venture between the Helmholtz Centre for Infection Research and the Hannover Medical School, Hannover, Germany; 9 Molecular Immunology, Helmholtz Centre for Infection Research, Braunschweig, Germany; 10 Institut für Klinische Chemie und Pathobiochemie, Klinikum rechts der Isar, Technische Universität München, Munich, Germany; 11 German Cancer Consortium (DKTK), Heidelberg, Germany; 12 German Center for Infection Research (DZIF), partner site Munich, Munich, Germany

## Abstract

The molecular pathways that regulate the tissue repair function of type I interferon (IFN-I) during acute tissue damage are poorly understood. We describe a protective role for IFN-I and the RIG-I/MAVS signaling pathway during acute tissue damage in mice. Mice lacking mitochondrial antiviral-signaling protein (MAVS) were more sensitive to total body irradiation– and chemotherapy-induced intestinal barrier damage. These mice developed worse graft-versus-host disease (GVHD) in a preclinical model of allogeneic hematopoietic stem cell transplantation (allo-HSCT) than did wild-type mice. This phenotype was not associated with changes in the intestinal microbiota but was associated with reduced gut epithelial integrity. Conversely, targeted activation of the RIG-I pathway during tissue injury promoted gut barrier integrity and reduced GVHD. Recombinant IFN-I or IFN-I expression induced by RIG-I promoted growth of intestinal organoids in vitro and production of the antimicrobial peptide regenerating islet–derived protein 3 γ (RegIIIγ). Our findings were not confined to RIG-I/MAVS signaling because targeted engagement of the STING (stimulator of interferon genes) pathway also protected gut barrier function and reduced GVHD. Consistent with this, STING-deficient mice suffered worse GVHD after allo-HSCT than did wild-type mice. Overall, our data suggest that activation of either RIG-I/MAVS or STING pathways during acute intestinal tissue injury in mice resulted in IFN-I signaling that maintained gut epithelial barrier integrity and reduced GVHD severity. Targeting these pathways may help to prevent acute intestinal injury and GVHD during allogeneic transplantation.

## Introduction

RIG-I belongs to the pattern recognition family of cytoplasmic RIG-I–like receptors. Its primary function is to detect double-stranded 5′-triphosphate RNA (3pRNA) during viral or bacterial infection (1–3). In contrast, the cytosolic DNA receptor cyclic guanosine monophosphate–adenosine monophosphate (cGAMP) synthase (cGAS) and its adapter protein STING (stimulator of interferon genes; TMEM173) recognize DNA in various contexts, for example, microbial DNA or nuclear DNA released into the cytosol by necrotic cells (4). Upon binding of ligand, RIG-I recruits the adaptor mitochondrial antiviral-signaling protein (MAVS) to induce proinflammatory cytokines, type I interferons (IFN-Is), and inflammasome activation (1, 5–8), orchestrating a diverse innate and adaptive immune response. cGAS binds to double-stranded DNA (dsDNA) and catalyzes the formation of cyclic dinucleotides. The latter can form cGAMP that activates STING to trigger innate immune gene transcription and IFN-I production (4). Whereas the role of IFN-I in initiating host defense against pathogens is well established, recent work highlights the regenerative function of this cytokine family, particularly at epithelial surfaces. IFN-I produced by plasmacytoid dendritic cells (pDCs) promotes skin repair upon mechanical barrier disruption (9) and increases intestinal epithelial turnover and repair of chemically damaged tissue. The effects of IFN-I on gut epithelial turnover have been attributed to both macrophage-dependent mechanisms (10) and Toll-like receptor stimulation of pDCs (11). However, the role of cytosolic nucleic acid sensors in this context is poorly understood. Similarly, the involvement of IFN-I in the repair of acute tissue damage by genotoxic insults has not been addressed. Unlike chemical injury of intestinal mucosa, irradiation- or chemotherapy-induced intestinal barrier dysfunction is a problem clinically.

Mucosal barriers like the intestinal epithelial cell (IEC) layer protect sterile microenvironments from physical, chemical, and microbial challenge. Epithelial integrity depends on constant and inducible IEC renewal by pluripotent intestinal stem cells (ISCs), which reside in the stem cell niche at the base of each intestinal crypt (12). Genotoxic stress by total body irradiation (TBI) or chemotherapy affects ISC and results in damage to the intestinal epithelium, ultimately causing translocation of microbes to sterile compartments and subsequent immune activation (13). During allogeneic hematopoietic stem cell transplantation (allo-HSCT), alteration of intestinal barrier function by chemotherapy or TBI administered before transplant has detrimental consequences: “Misplaced” bacterial components together with endogenous danger signals released during epithelial cell death are sensed by pattern recognition receptors on antigen-presenting cells, which then produce proinflammatory cytokines and prime donor–derived T cells (13). These alloreactive T cells attack and destroy host tissues, primarily the gastrointestinal tract, liver, and skin, causing morbidity and mortality in a process called acute graft-versus-host disease (GVHD). GVHD is the leading complication after allo-HSCT and occurs in as many as 50% of transplant recipients.

Thus, investigating molecular mechanisms that promote intestinal epithelial integrity and repair during tissue injury is fundamental to the development of new approaches to prevent treatment-associated inflammation and GVHD. The RIG-I/MAVS and STING signaling pathways are important regulators of IFN-I production, and IFN-I can initiate epithelial repair. Thus, we hypothesized that activation of these pathways during pretransplant bone marrow (BM) ablative therapy in mice and during allo-HSCT may protect epithelial integrity and could be exploited to promote intestinal barrier function and prevent GVHD.

## Results

### Endogenous RIG-I/MAVS signaling reduces intestinal tissue damage induced by conditioning therapy and attenuates GVHD in mice

**Fig. 1:**
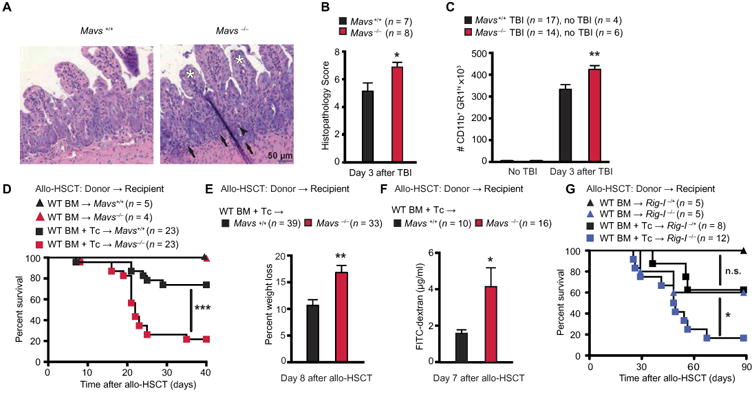
Endogenous RIG-I/MAVS signaling reduces intestinal tissue damage in mice (**A**) Representative images of tissue damage in hematoxylin and eosin–stained small intestine biopsies from mice after an 11-Gy TBI conditioning regimen. White asterisks, villus stunting; black arrowhead, crypt apoptosis; black arrows, granulocyte infiltration. (**B**) Histopathological score from (A); pooled data from two independent experiments. (**C**) Number of leukocytes infiltrating the mouse gut lamina propria after TBI (11 Gy) analyzed by flow cytometry. Pooled data from four independent experiments. Survival (**D**) and weight loss (**E**) in mouse allo-HSCT recipients transplanted with 5 × 10^6^ BM ± 2 × 10^6^ T cells (donor BALB/c WT and C57BL/6 *MAVS*^+/+^ or *MAVS*^−/−^ recipients). Pooled data from four independent experiments. (**F**) FITC-dextran concentrations in plasma after allo-HSCT [described in (D) and (E)]. Pooled data from two independent experiments. (**G**) Survival of *Rig-I*^−/+^ and *Rig-I*^−/−^ mouse recipients after transplant with 5 × 10^6^ BM and 1 × 10^6^ T cells (donor C57BL/6 WT, recipient 129/sv *Rig-I*^−/+^ or *Rig-I*^−/−^). Animal numbers per group (*n*) are depicted in the figure panels. Survival was analyzed using the log-rank test. All other experiments were analyzed using two-tailed unpaired *t* test. *P < 0.05, **P < 0.01, and ***P < 0.001. Data are means ± SEM. n.s., not significant.

We first assessed genotoxic tissue damage and regeneration in wild-type (WT) mice and mice genetically deficient in MAVS (*Mavs*^−/−^). Mice were exposed to lethal TBI, which caused damage to dividing cells and induced loss of intestinal epithelial barrier function (14, 15). Compared to *Mavs*^+/+^ littermates, *Mavs*^−/−^ mice exhibited worse mucosal damage in the small intestine with increased crypt apoptosis, villus atrophy, crypt abscesses, and granulocytic infiltrates (Fig. 1, A and B). Neutrophil influx into the gut mucosa, a surrogate marker for intestinal integrity (16), was higher in *Mavs*^−/−^ compared to *Mavs*^+/+^ littermates after TBI (Fig. 1C) or chemotherapy with doxorubicin (fig. S1A) (17). Consequently, in an acute GVHD model, where conditioning-associated intestinal damage is crucial for subsequent allogeneic T cell–mediated pathology, we observed that *Mavs*^−/−^ recipients of allogeneic donor BM and T cells had increased mortality compared to *Mavs*^+/+^ littermates (Fig. 1D). In addition, Mavs^−/−^ allo-HSCT recipients exhibited greater weight loss (Fig. 1E and fig. S1, B and C) and reduced intestinal barrier integrity as measured by translocation of intraluminal fluorescein isothiocyanate (FITC)–dextran into the systemic circulation on day 7 after allogeneic transplantation (Fig. 1F and fig. S1D). Rig-I^−/−^ (*Ddx58*^−/−^) allo-HSCT recipients of donor BM and T cells also displayed increased mortality and weight loss compared to Rig-I^+/−^ littermates (Fig. 1G and fig. S1E). We observed a nonsignificant trend toward higher mortality and more weight loss of Rig-I^−/−^ recipients of allogeneic donor BM (Fig. 1G and fig. S1E).

### MAVS signaling in nonhematopoietic cells maintains intestinal barrier function and attenuates GVHD in mice

**Fig. 2:**
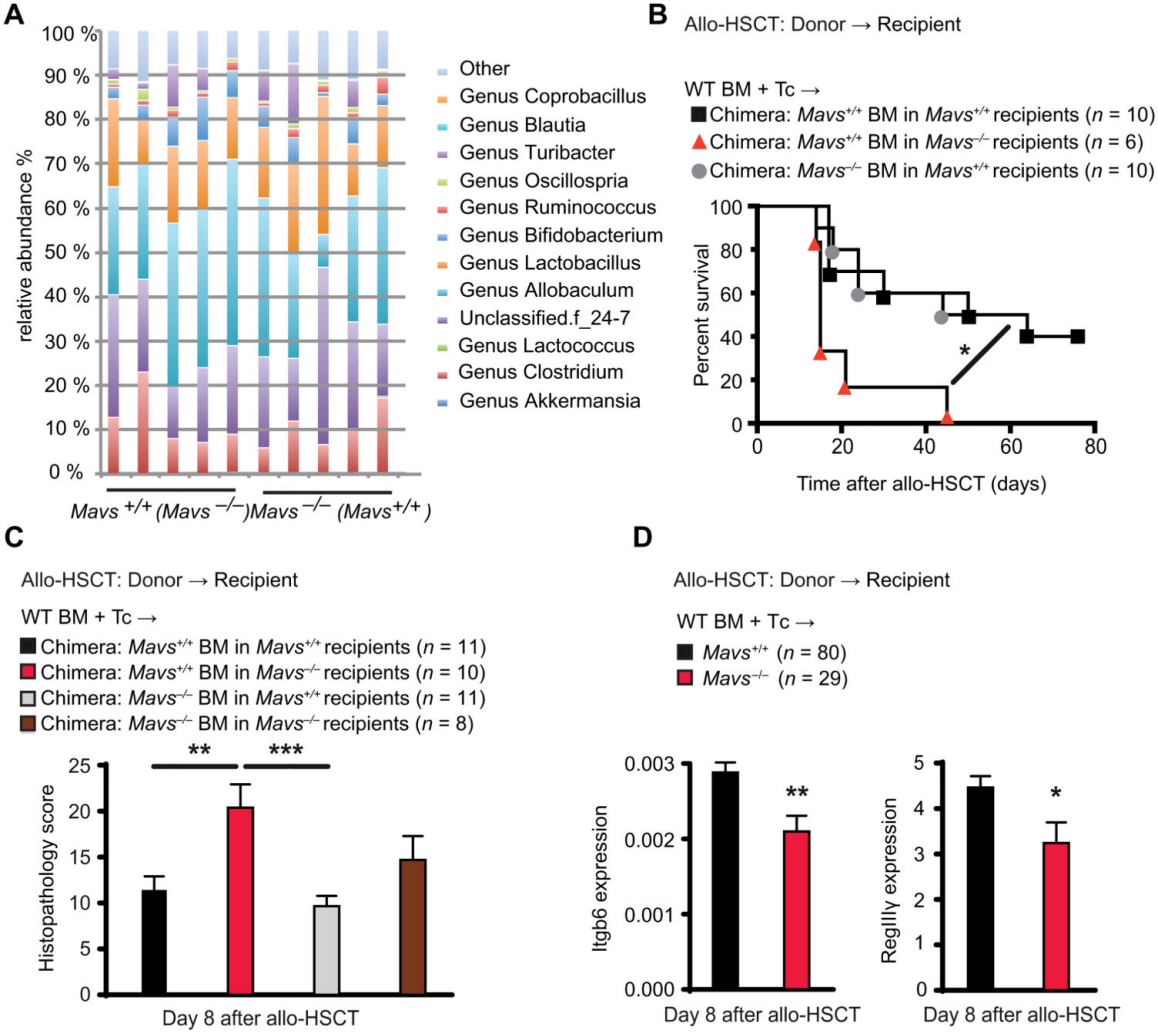
MAVS signaling in nonhematopoietic cells maintains intestinal barrier function (**A**) Average relative abundance of bacterial genera in the intestinal microbiota of cohoused *Mavs*^+/+^ (*n* = 5) and *Mavs*^−/−^ (*n* = 5) litter-mates. One representative experiment of two independent experiments. (**B**) Survival of C57BL/6 BM chimeric mice, which were Mavs-deficient either in the hematopoietic or in the nonhematopoietic compartment. These mice were analyzed after a second allo-HSCT with BM and T cells from B10.BR WT donor mice. (**C**) GVHD histopathological score for small intestine biopsies from C57BL/6 BM chimeric mice, which were Mavs-deficient either in the hematopoietic or in the nonhematopoietic compartment. These mice were analyzed after a second allo-HSCT with BM and T cells from BALB/c WT donor mice. Pooled data from two independent experiments. (**D**) Quantitative polymerase chain reaction (qPCR) of *Itgb6* and *RegIII*γ expression in the small intestine after allo-HSCT with BM and T cells from BALB/c WT mouse donors into C57BL/6 *MAVS*^+/+^ or *MAVS*^−/−^ recipients. Pooled data from five independent experiments. Animal numbers per group (*n*) are depicted above panels. Survival was analyzed using the log-rank test. Other experiments were analyzed using ordinary one-way analysis of variance (ANOVA) for multiple comparisons or two-tailed unpaired *t* test. *P < 0.05, **P < 0.01, and ***P < 0.001. Data are means ± SEM.

Given that the RIG-I/MAVS pathway senses bacterial RNA (18), one hypothesis to explain our findings is that there may be mouse strain–specific differences in the intestinal bacterial microbiota. We could not detect differences between the intestinal bacterial composition of co-housed *Mavs*^−/−^ and *Mavs*^+/+^ littermates as assessed by 16S ribosomal RNA (rRNA) sequencing (Fig. 2A). To define the effects of RIG-I/MAVS deficiency in a compartment-specific manner, we generated BM chimeras with either a MAVS-deficient or MAVS-sufficient hematopoietic system or non-hematopoietic system, respectively. This approach yielded donor chimerism of >99% among intestinal myeloid cells (fig. S2A). BM chimeras with MAVS deleted in the nonhematopoietic system (MAVS^+/+^ BM transplanted into *Mavs*^−/−^ recipients) showed higher mortality after allo-HSCT (Fig. 2B) and more intestinal pathology in the small intestine (Fig. 2C) compared to WT recipients of WT BM (MAVS^+/+^ BM transplanted into *Mavs*^+/+^ recipients) or WT recipients with MAVS deleted in the hematopoietic system (MAVS^−/−^ BM transplanted into *Mavs*^+/+^ recipients). We next analyzed gene expression of integrin β_6_ (*Itgb6*) in small intestine RNA isolates as an indicator of epithelial cell integrity after damage (19, 20) and of the antimicrobial peptide RegIIIγ, which is produced by Paneth cells and protects the inner mucus layer from bacterial colonization (21). Both Itgb6 and RegIIIg gene expressions were reduced in *Mavs*^−/−^ allo-HSCT recipients compared to *Mavs*^+/+^ littermates (Fig. 2D). Because reduced gut epithelial barrier function may promote allogeneic T cell reactivity, we next analyzed donor-derived CD4 and CD8 T cell expansion and IFN-γ production of *Mavs*^+/+^ versus *Mavs*^−/−^ allo-HSCT recipient mice. We observed increased T cell proliferation in the spleen of *Mavs*^−/−^ recipients early after allo-HSCT (day 4 after transplant) but similar T cell effector function at later time points in the small intestine (day 8 after transplant) (fig. S2, B and C).

### RIG-I/MAVS pathway activation protects mice from intestinal tissue damage after conditioning therapy

**Fig. 3:**
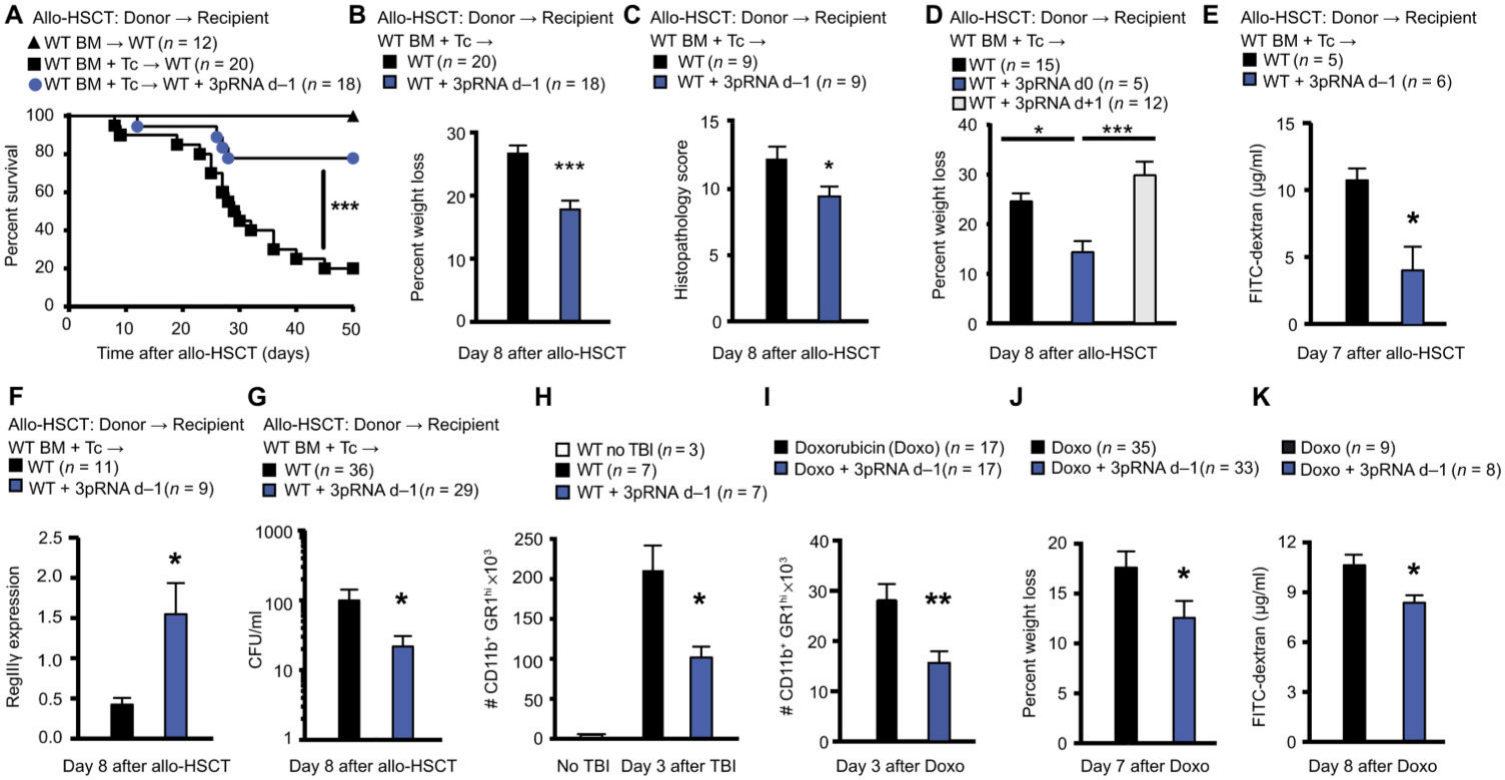
RIG-I/MAVS pathway activation protects from intestinal tissue damage after TBI Survival (**A**) and weight loss (**B**) after allo-HSCT involving transplant of 5 × 10^6^ BM ± 1 × 10^6^ T cells from C57BL/6 WT donor mice into BALB/c WT recipients with or without 3pRNA treatment on day −1. Pooled data from four independent experiments. (**C**) Histopathological score of small intestine tissue after allo-HSCT from C57BL/6 WT donor mice to BALB/c WT mouse recipients with or without 3pRNA treatment on day −1. Pooled data from two independent experiments. (**D**) Weight loss in mouse recipients from (C) after allo-HSCT with or without 3pRNA treatment on day 0 (d0) or day +1 (d+1). Pooled data from three independent experiments. (**E**) FITC-dextran concentrations in plasma after allo-HSCT from C57BL/6 WT donor mice to BALB/c WT recipient mice with or without 3pRNA treatment on day −1. (**F**) qPCR of *RegIII*γ expression in mouse gut epithelial cells after allo-HSCT from C57BL/6 WT donor mice into BALB/c WT recipient mice with or without 3pRNA treatment on day −1. Pooled data from three independent experiments. (**G**) Bacterial colony-forming units (CFU) in sera from mouse recipients after allo-HSCT [described in (F)]. Pooled data from three independent experiments. (**H**) Leukocytes in the small intestine lamina propria of BALB/c mice after TBI (9 Gy) analyzed by flow cytometry. Pooled data from two independent experiments. (**I**) Leukocytes in the small intestine lamina propria of C57BL/6 mice after treatment with chemotherapy (doxorubicin) analyzed by flow cytometry. Pooled data from three independent experiments. (**J**) Weight loss in C57BL/6 mice that received doxorubicin (20 mg/kg). Pooled data from six independent experiments. (**K**) FITC-dextran concentrations in plasma from C57BL/6 mice after doxorubicin treatment (20 mg/kg). One representative experiment of four independent experiments is shown. Animal numbers per group (n) are depicted above figure panels. 3pRNA treatment was always performed on day −1 in indicated groups except for (D). All experiments were analyzed using one-tailed (G) or two-tailed unpaired *t* test or ordinary one-way ANOVA for multiple comparisons. Survival was analyzed using the log-rank test. *P < 0.05, **P < 0.01, and ***P < 0.001. Data are means ± SEM.

We observed that a single dose of intravenous 3pRNA, a RIG-I agonist, 1 day before allo-HSCT reduced mortality (Fig. 3A), weight loss (Fig. 3B and fig. S3A), and damage to the small intestine compared to control WT recipients who did not receive 3pRNA (Fig. 3C and fig. S3B). It was critical that RIG-I agonists were administered before (day −1) or at the same time as allo-HSCT (day 0), given that administration after allo-HSCT (day +1) failed to achieve a benefit (Fig. 3D and fig. S3C). Non-triphosphorylated RNA that does not activate RIG-I (22) did not reduce weight loss or improve survival (fig. S3D). This suggested that activation of the RIG-I/MAVS pathway protects from intestinal damage. Administration of 3pRNA (day −1) led to decreased gut mucosal permeability as measured by FITC-dextran translocation (Fig. 3E) and to enhanced intestinal expression of RegIIIγ in the small intestine after allo-HSCT (Fig. 3F). Similarly, pretreatment with 3pRNA lowered systemic bacteremia after allo-HSCT (Fig. 3G) and reduced neutrophil infiltration after TBI (Fig. 3H) but did not result in altered production of the proinflammatory cytokines interleukin-6 (IL-6) and tumor necrosis factor–α compared to TBI alone (fig. S3E). 3pRNA treatment before barrier-disrupting chemotherapy with doxorubicin also reduced neutrophil infiltration and decreased weight loss and translocation of FITC-dextran (Fig. 3, I to K, and fig. S3F). Consistent with the concept that avoiding breaching the epithelial barrier could prevent GVHD, 3pRNA pretreatment reduced allogeneic T cell activation in the spleen and intestine of allo-HSCT recipient WT mice (fig. S3, G and H). Decreased allogeneic T cell activity and GVHD may be accompanied by a reduction in the beneficial graft-versus-leukemia (GVL) response. However, application of 3pRNA on day -1 before allo-HSCT along with A20-Luc–transduced lymphoma cells did not diminish GVL activity against the latter compared to control allo-HSCT recipient WT mice who did not receive 3pRNA (fig. S3, I and J).

### RIG-I–induced type I IFN signaling mediates intestinal tissue protection and prevents GVHD in mice

**Fig. 4:**
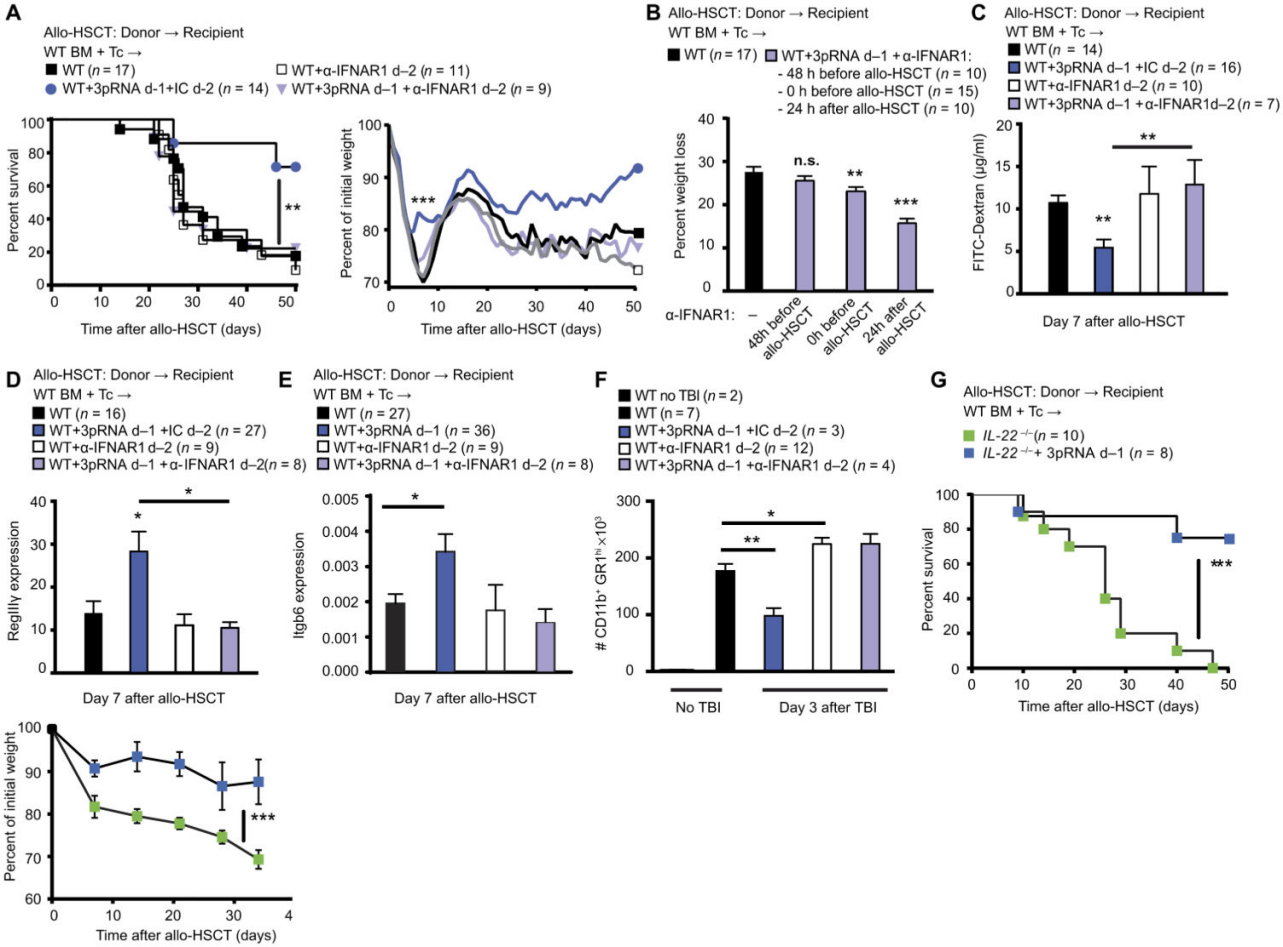
RIG-I–induced type I IFN signaling mediates intestinal tissue protection (**A**) Survival and percent of initial weight after allo-HSCT. BM cells (5 × 10^6^) plus T cells (1 × 10^6^) from C57BL/6 WT mice were transplanted into BALB/c recipient mice with or without 3pRNA treatment on day −1 before allo-HSCT and treatment with IFNAR1-blocking antibody (α-IFNAR1) or immunoglobulin G1 (IgG1) isotype control on day −2 before allo-HSCT. Pooled data from three independent experiments. (B) Weight loss after allo-HSCT. BM cells (5 × 10^6^) plus T cells (1 × 10^6^) from C57BL/6 WT mice were transplanted into BALB/c recipient mice with or without 3pRNA treatment on day −1 before allo-HSCT and treatment with IFNAR1-blocking antibody (α-IFNAR1) 48 hours before allo-HSCT (−48 h before TX), at the time of allo-HSCT (0 h before TX), or 24 hours after allo-HSCT (24 h after TX). (**C**) FITC-dextran concentrations in plasma of BALB/c mouse recipients after allo-HSCT, with or without 3pRNA treatment on day −1 before allo-HSCT, and treatment with IFNAR1-blocking antibody (α-IFNAR1) or IgG1 isotype control (IC) on day −2 before allo-HSCT. Pooled data from three independent experiments. (**D** and **E**) qPCR of *RegIII*γ and *Itgb6* expression in small intestine biopsies from BALB/c recipient mice after allo-HSCT, with or without 3pRNA treatment on day −1 before allo-HSCT, and treatment with IFNAR1-blocking antibody (α-IFNAR1) or IgG1 isotype control on day −2 before allo-HSCT. Pooled data from three (D) and six (E) independent experiments. (**F**) Flow cytometry analysis of leukocyte infiltration into the small intestine lamina propria of BALB/c mice after TBI(9 Gy), with or without 3pRNA treatment on day −1 before TBI, and treatment with IFNAR1-blocking antibody (α-IFNAR1) or IgG1 isotype control on day −2 before TBI. Pooled data from two independent experiments. (G) Survival and weight loss in BALB/c mouse recipients lacking IL-22 (*Il-22*^−/−^) after allo-HSCT with BM and T cells from C57BL/6 WT mice with or without 3pRNA treatment on day −1 before allo-HSCT. Pooled data from two independent experiments. Animal numbers per group (n) are depicted above figure panels. Treatment time points for 3pRNA and α-IFNAR1 antibody are indicated. All experiments were analyzed using two-tailed unpaired *t* test or ordinary one-way ANOVA for multiple comparisons. Survival was analyzed using the log-rank test. *P < 0.05, **P < 0.01, and ***P < 0.001. Data are means ± SEM.

We next analyzed the role of IFN-I in intestinal tissue protection and prevention of GVHD. Systemic application of 3pRNA led to a rapid increase in IFN-α and IFN-β in the serum (fig. S4A), enhanced IFN-β luciferase reporter activity in the intestine of IFN-β luciferase reporter mice (fig. S4B) (22), and increased expression of IFN-induced genes, including RIG-I (*Ddx58*) and *Mx1*, in IECs isolated from the small intestine (fig. S4C). We then performed RNA sequencing with tissue samples from the small intestine of WT mice that received TBI before 3pRNA treatment and antibody-mediated blockade of the IFN-α/β receptor (IFNAR) (fig. S4D). 3pRNA treatment before TBI resulted in increased expression of IFN-inducible genes in the small intestine. Blockade of IFNAR signaling abrogated 3pRNA-mediated up-regulation of IFN-induced genes, demonstrating that RIG-I–induced gene regulation depends on IFN-I. Upon temporary blockade of IFNAR signaling directly before 3pRNA treatment, RIG-I agonists failed to improve overall mouse survival and early weight loss after allo-HSCT (Fig. 4A). This indicated that RIG-I agonists required IFN-I signaling to be protective. In line with our findings with 3pRNA (Fig. 3, B and D, and fig. S3C), the induction of IFN-I was only effective before TBI-induced damage. Blocking IFNAR 48 hours before tissue damage abrogated the effects of 3pRNA, whereas blockade of IFNAR 24 hours after damage did not (Fig. 4B and fig. S4E). Consistent with these results, blockade of IFN-I signaling 2 days before TBI-induced damage abrogated the 3pRNA-induced increase in barrier function (Fig. 4C) and the increase in *RegIII*γ and *Itgb6* expression during GVHD (Fig. 4, D and E), and reversed the inhibition of neutrophil influx into the gut mucosa after TBI-induced intestinal damage in WT mice (Fig. 4F). Notably, recipient-derived IL-22 has been shown to protect against tissue damage caused by conditioning therapy and GVHD (23, 24) by protecting intestinal epithelial integrity. However, 3pRNA was effective in reducing GVHD and weight loss in *IL-22*^−/−^ allo-HSCT recipient mice (Fig. 4G). This suggested that IFN-I, but not IL-22, may be the mediator of RIG-I–induced protection.

### RIG-I–induced type I IFN signaling in nonhematopoietic cells promotes proliferation of the ISC compartment

**Fig. 5:**
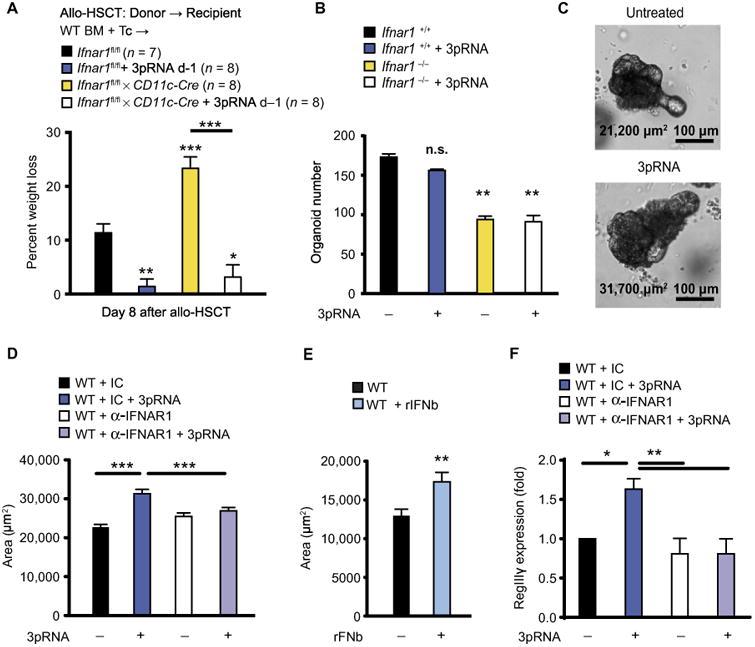
RIG-I–induced type I IFN signaling in nonhematopoietic cells promotes regeneration of the ISC compartment (**A**) Weight loss after allo-HSCT. BM cells (5 × 10^6^) plus T cells (5 × 10^6^) from BALB/c donor mice transplanted into C57BL/6 recipient mice (genotypes are indicated in the figure) with or without 3pRNA treatment on day −1. Pooled data from two independent experiments. (**B**) Number of organoids after 5 days in culture with or without the addition of 3pRNA (2 μg/ml) on day 1 of culture; organoids were derived from C57BL/6 *IFNAR1*^+/+^ or *IFNAR1*^−/−^ mice. One representative experiment of three independent experiments. (**C**) Representative images of organoids derived from C57BL/6 WT mice after 5 days in culture with or without the addition of 3pRNA (2 μg/ml) on day 1 of culture. Organoid area is shown. (**D**) Measurement of organoids from C57BL/6 WT mice after 5 days in culture with or without the addition of 3pRNA (2 μg/ml) or a-IFNAR1–blocking antibody (10 μg/ml) on day 1 of culture. One representative experiment of three independent experiments. (**E**) Size of organoids derived from C57BL/6 WT mice after 7 days in culture with or without the addition of recombinant murine IFN-b (20 U/ml) on day 1 of culture. One representative experiment of three independent experiments. (**F**) qPCR of *RegIII*γ expression in organoids 24 hours after stimulation with indicated combinations of 3pRNA and α-IFNAR1–blocking antibody or IgG1 isotype control. Pooled data from three independent experiments. All experiments were analyzed using two-tailed unpaired t test or ordinary one-way ANOVA for multiple comparisons. Survival was analyzed using the log-rank test. *P < 0.05, **P < 0.01, and ***P < 0.001. Data are means ± SEM.

The observed increase in gut barrier function and increased IFN-β production in the intestine after 3pRNA administration (fig. S4B) suggested a prominent role for the nonhematopoietic system including IECs as IFN-I targets in vivo. We therefore generated BM chimeric mice with either an IFNAR1 (IFN-α/β receptor α-chain)–deficient hematopoietic compartment or an IFNAR1-deficient nonhematopoietic compartment and used them as allo-HSCT recipients with or without previous 3pRNA treatment. Mice with IFNAR1 deficiency in the nonhematopoietic system developed more severe GVHD than did those with IFNAR1 deficiency in the hematopoietic system (fig. S5A). There was a nonstatistically significant trend toward improved survival after RIG-I agonist treatment in allo-HSCT recipients with an IFNAR1-deficient hematopoietic system but not in allo-HSCT recipients with an IFNAR1-deficient nonhematopoietic system (fig. S5A). Although these data may suggest a prominent role for IFNAR1, in the nonhematopoietic system, the origin and target of IFNs produced in vivo remain unclear. DCs are a main hematopoietic target population of IFN-I activity in vivo (25), which prompted us to analyze the effects of 3pRNA on the course of GVHD in mice in which CD11c^+^ DCs did not express IFNAR1. We found more early weight loss during GVHD in *CD11cCre Ifnar1^fl/fl^* mice, compared to cohoused *Ifnar1^fl/fl^* mice (Fig. 5A), whereas overall survival was not significantly different (fig. S5B). However, 3pRNA-mediated prevention of early weight loss was unchanged (Fig. 5A), confirming a predominant role of the nonhematopoietic system in mediating the effects of IFN-I. We thus postulated that prophylactic RIG-I−triggered protection from tissue injury could be mediated by IFN signaling in IECs. To assess the direct impact of RIG-I signaling and IFN-I on IECs, we used an ex vivo organoid system composed of mouse primary small intestine crypts (26). Each of these epithelial “mini-guts” contained a functional ISC compartment that consisted of LGR5^+^ ISCs and supportive niche cells (Paneth cells) (26). Crypts cultured ex vivo grew into organoids with crypt buds that recapitulated the in vivo intestinal organization including crypt villus structures and central lumen markers (27). Fewer epithelial organoids were derived from *Ifnar1*^−/−^ compared to *Ifnar1*^+/+^ mice, suggesting a crucial role for type I IFN signaling in epithelial regeneration (Fig. 5B). Although ex vivo stimulation with 3pRNA did not increase the number of intestinal organoids (Fig. 5B), it did increase organoid size (Fig. 5, C and D, and fig. S5C), suggesting that RIG-I activation stimulated the ISC compartment, resulting in epithelial tissue regeneration.

**Fig. 6:**
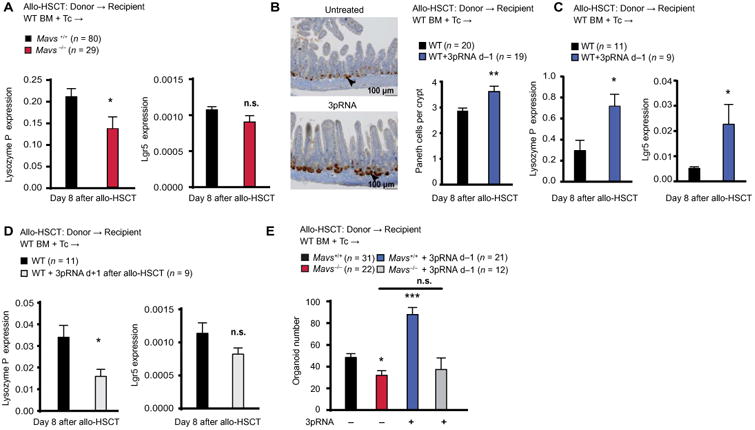
RIG-I activation protects ISCs after allo-HSCT (**A**) qPCR expression of *Lysozyme P* and *Lgr5* in small intestine biopsies from *MAVS*^+/+^ or *MAVS*^−/−^ C57BL/6 recipient mice after allo-HSCT with 5 × 10^6^ BM cells and 2 × 10^6^ T cells from BALB/c WT donor mice. Pooled data from five independent experiments. (**B**) Analysis of allo-HSCT BALB/c recipients in the presence or absence of 3pRNA treatment on day −1. Immunohistochemistry showing lysozyme staining of the small intestine of BALB/c recipients 8 days after allo-HSCT. Lysozyme-positive gut Paneth cells are indicated by black arrowheads. Histogram shows number of Paneth cells per crypt. Representative images and pooled data from three independent experiments. (**C**) qPCR showing expression of *Lysozyme P* and *Lgr5* in IECs from the small intestine of BALB/c recipient mice 8 days after allo-HSCT with or without 3pRNA treatment on day −1 before allo-HSCT. Pooled data from three independent experiments. (**D**) qPCR of *Lysozyme P* and *Lgr5* expression in the small intestine of BALB/c recipient mice after allo-HSCT with or without 3pRNA treatment on day +1. Data from one experiment. (**E**) Number of organoids derived from C57BL/6 recipient mice on day 8 after allo-HSCT with or without 3pRNA treatment on day −1 before allo-HSCT. Pooled data from four independent experiments. Animal numbers per group (*n*) are depicted above figure panels. All experiments were analyzed using two-tailed unpaired *t* test or ordinary one-way ANOVA for multiple comparisons. *P < 0.05, **P < 0.01, and ***P < 0.001. Data are means ± SEM.

Similar to our in vivo findings demonstrating that 3pRNA-induced augmentation of gut barrier function was mediated by IFNAR signaling (Fig. 4, C to F), organoid growth after ex vivo 3pRNA stimulation was dependent on IFN-I produced via MAVS (fig. S5D). IFNAR blockade abrogated 3pRNA-mediated increase in organoid size (Fig. 5D and fig. S5C), whereas addition of recombinant IFN-β increased organoid size (Fig. 5E and fig. S5E). Neither recombinant IFN-β nor IFNAR blockade could influence the number of organoids (fig. S5, F and G). Yet, 3pRNA stimulation of organoid-forming crypts ex vivo also induced IFN-I–dependent RegIIIγ expression (Fig. 5F), consistent with our in vivo findings (Fig. 4D). Unlike the case with *IFNAR*^−/−^ organoids, we could not detect an inherent difference in organoid formation between small intestine crypts isolated from *Mavs*^+/+^ or *Mavs*^−/−^ littermates (fig. S6A). Furthermore, no differences in Paneth cell numbers between *Mavs*^+/+^ and *Mavs*^−/−^ littermates could be detected (fig. S6B), suggesting that MAVS signaling in the intestine may not be required for mediating gut homeostasis in the steady state but is required for the induction of epithelial regeneration after tissue damage. We found that the Paneth cell–derived antimicrobial peptide Lysozyme P and the ISC marker Lgr5 were both reduced in *Mavs*^−/−^ mice compared to *Mavs*^+/+^ litter-mates after allo-HSCT (Fig. 6A). We found elevated numbers of Paneth cells in allo-HSCT recipients pretreated with 3pRNA on day −1 (Fig. 6B) and elevated gene expression of Lysozyme P and the ISC marker Lgr5 (Fig. 6C). Congruent with a lack of benefit for GVHD, we found that delaying 3pRNA treatment until day +1 after allo-HSCT did not protect ISCs and Paneth cells. *Lysozyme P* and *Lgr5* expression was reduced in mice that received 3pRNA on day +1 after allo-HSCT (Fig. 6D). We also observed that *Lysozyme P* and *Lgr5* expression was reduced in mice 24 hours after TBI, suggesting that the lack of efficacy of 3pRNA after allo-HSCT could at least in part be due to fewer target cells in the intestinal epithelium (fig. S6C).

To determine the impact of endogenous RIG-I/MAVS signaling on intestinal regeneration during ongoing GVHD, we next analyzed the capacity to form intestinal organoids ex vivo in *Mavs*^+/+^ compared to *Mavs*^−/−^ allo-HSCT recipients. Strikingly, fewer organoids could be retrieved from *Mavs*^−/−^ allo-HSCT recipients than from *Mavs*^+/+^ litter-mates (Fig. 6E). We also found that in vivo 3pRNA treatment before (day −1) allo-HSCT induced more organoid growth compared to untreated WT recipients. This effect was absent in *Mavs*^−/−^ allo-HSCT recipients (Fig. 6E), demonstrating that 3pRNA engages the RIG-I/MAVS pathway in vivo to exert its protective function.

### STING signaling protects allo-HSCT recipients from GVHD and regulates intestinal organoid growth

**Fig. 7:**
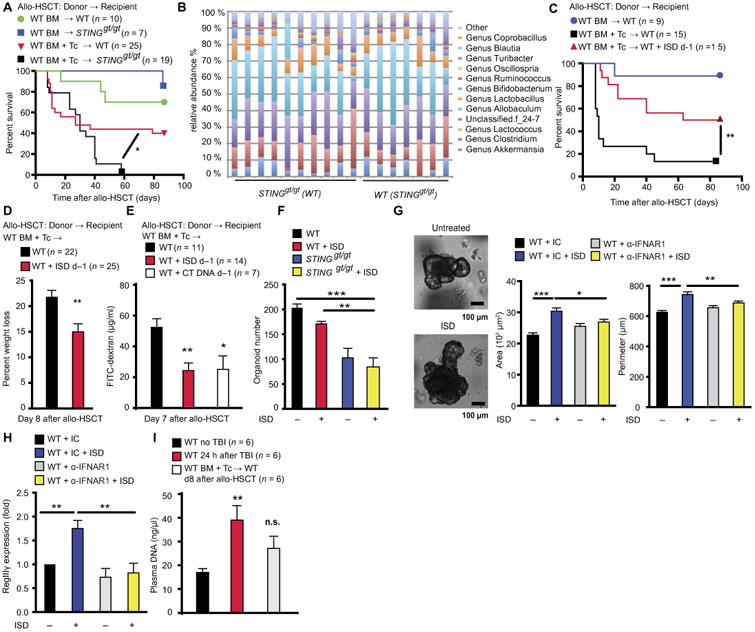
STING pathway protects mouse recipients from GVHD after allo-HSCT (**A**) Survival of cohoused C57BL/6 WT or *Sting^gt/gt^* mice that received 5 × 10^6^ BM cells plus1× 10^6^ T cells from WT B10.BR donor mice. Pooled data from two independent experiments. (**B**) Relative abundance of bacterial genera in the intestinal microbiota of cohoused WT and *Sting^gt/gt^* mice. One representative experiment of two independent experiments. Survival (**C**) and weight loss (**D**) for C57BL/6 mouse recipients after allo-HSCT from B10.BR donor mice with or without IFN-stimulatory DNA (ISD) treatment on day −1. Pooled data of two (C) or three (D) independent experiments. (**E**) FITC-dextran concentrations in plasma of BALB/c mouse recipients after allo-HSCT from C57BL/6 donors with or without calf thymus DNA (CT DNA) or IFN-stimulatory DNA treatment on day −1. Pooled data from two independent experiments. (**F**) Number of organoids derived from WT or *Sting^gt/gt^* C57BL/6 mouse small intestine after 5 days in culture with or without the addition of IFN-stimulatory DNA (2 μg/ml) on day 1 of culture. Pooled data from three independent experiments. (**G**) Organoids derived from C57BL/6 mouse small intestine. Measurement of organoid size after 5 days in culture with or without the addition of IFN-stimulatory DNA (2 μg/ml), α-IFNAR1–blocking antibody (10 μg/ml), or IgG1 isotype control on day 1 of culture. The experiment was performed three times, and images of one representative experiment are shown. (**H**) qPCR of *RegIII*γ expression in organoids derived from C57BL/6 WT mice 24 hours after stimulation with indicated combinations of IFN-stimulatory DNA, α-IFNAR1–blocking antibody, or IgG1 isotype control. Pooled data from three independent experiments. (I) DNA in plasma of BALB/c mice 24 hours after TBI (9 Gy) or allo-HSCT. Pooled data from three independent experiments. Animal numbers per group (*n*) are depicted above figure panels. All experiments were analyzed using two-tailed unpaired *t* test or ordinary one-way ANOVA for multiple comparisons. Survival was analyzed using the log-rank test. *P < 0.05, **P < 0.01, and ***P < 0.001. Data are means ± SEM.

Considering the protective role of RIG-I/MAVS against genotoxic tissue damage, we wondered whether other IFN-I–inducing cytosolic nucleic acid sensors would have similar effects. We therefore transplanted STING Goldenticket (*Sting^gt/gt^*) mice with allogeneic BM and T cells. Similar to our observation in *Mavs*^−/−^ mice, we found that *Sting^gt/gt^* allo-HSCT recipients showed increased mortality compared to cohoused WT mice (Fig. 7A). The composition of the gut microbiota was comparable between cohoused WT and *Sting^gt/gt^* mice (Fig. 7B). In parallel with our 3pRNA results, we found that allo-HSCT recipients treated with IFN-stimulatory DNA on day −1 showed reduced mortality (Fig. 7C) and weight loss (Fig. 7D). Administration of IFN-stimulatory DNA induced production of IFN-α and IFN-β in serum and, like 3pRNA, did not change TBI-induced production of proinflammatory cytokines (fig. S7, A and B). Administration of IFN-stimulatory DNA also reduced translocation of FITC-dextran across the mouse gut epithelia. This effect could be reproduced after injection of calf thymus DNA, which, in contrast to IFN-stimulatory DNA, contained CpG motifs (28) and induced IFN-I via the STING pathway (Fig. 7E) (29). Finally, STING signaling and stimulation of organoids with IFN-stimulatory DNA contributed to growth of intestinal organoids in vitro and *RegIII*γ expression in an IFN-I–dependent manner (Fig. 7, F to H).

Given that pretransplant conditioning with either TBI or chemotherapy leads to accumulation of aberrant self-DNA found in apoptotic bodies, the extracellular space, and cytosol resulting in IFN-I production (30), we hypothesized that the STING pathway might mediate protection through detection of endogenous DNA. We observed increased dsDNA in the plasma of mice undergoing TBI compared to untreated mice (Fig. 7I). We next used the luciferase reporter mouse system to analyze IFN-β production in the intestine 24 hours after TBI. In comparison to untreated mice, TBI induced IFN-I signaling in the small intestine (fig. S7C).

We did not succeed in detecting endogenous RNA in mouse plasma. If endogenous RNA was released into the extracellular space upon damage, then we presumed it was rapidly degraded. Given that commensal microbiota including bacteria in the gut could potentially deliver endogenous ligands required for activation of RIG-I (18), we tested whether RNA isolated from mouse feces could induce RIG-I–dependent IFN-I signaling in IECs. Feces-derived RNA induced a RIG-I–dependent IFN-I response in MODE-K cells, a murine IEC line with morphological and phenotypic characteristics of normal enterocytes (31), arguing that 3pRNA and RNA derived from commensals including viruses, phage, or bacteria could potentially induce protective IFN-I signaling through activation of RIG-I (fig. S7, D to G).

Together, our data suggest that activation of the RIG/MAVS and STING pathways, either through endogenous or applied ligands (IFN-stimulatory DNA, 3pRNA), may be essential for protection of gut epithelial integrity after TBI or chemotherapy and for the prevention of GVHD after allo-HSCT.

## Discussion

Previous studies have proposed a protective function of IFN-I in the setting of allo-HSCT (32) and of stromal MAVS signaling in a dextran sodium sulfate (DSS)–induced mouse model of colitis (18), but the mechanisms by which IFN-I contributes to this protection remain ill defined. Li and co-workers (18) used a model of low-dose DSS to induce chronic tissue damage and demonstrated that MAVS signaling in stromal cells controlled tissue homeostasis by monitoring commensal bacteria. However, erosive epithelial damage by DSS is an artificial experimental approach that does not mirror common clinical scenarios, in which patients suffer from tissue damage after cytotoxic chemotherapy or radiation therapy or through immune activation. Here, we have used a series of genetically modified (*Ddx58*^−/−^, *Mavs*^−/−^, and Sting^*gt/gt*^) and chimeric mice to analyze clinically relevant models of injury (TBI and chemotherapy) to the ISC compartment and immune-mediated acute tissue damage (allo-HSCT/GVHD). We have demonstrated the role of the RIG-I/MAVS/IFN-I and STING/IFN-I pathways for the maintenance of intestinal barrier function and prevention of GVHD. Specifically, we have shown that defective MAVS or STING signaling leads to breakdown of intestinal barrier function and increased GVHD pathology. Given that cohoused WT and *Mavs*^−/−^ or *Sting^gt/gt^* mice harbored similar intestinal bacterial populations, it is unlikely that differences in bacterial composition contributed to the protective role of MAVS or STING during GVHD development, unlike what has been proposed for IFN-I–mediated control of Paneth cell function (33). *Rig-I*^−/−^ mouse recipients of allogeneic BM and T cells similarly suffered from worse GVHD, and there was a nonsignificant trend toward higher mortality and more weight loss in *Rig-I*^−/−^ mouse recipients receiving allogeneic BM only. This is reminiscent of the colitis-like phenotype of *Rig-I*^−/−^ mice and their increased susceptibility to DSS-induced colitis (34). However, because *Rig-I*^−/−^ mice seemed to be viable only on the 129/sv background, their higher susceptibility to external insults may be attributable to strain-specific differences.

We showed that exogenous stimulation of the RIG-I and STING pathways with 3pRNA or IFN-stimulatory DNA in a preventive setting (1 day before allo-HSCT) promoted intestinal barrier function (as measured by FITC-dextran translocation), Paneth cell function (measured by expression of *Lysozyme P*), *Lgr5* marker expression, and the production of mucosal homeostatic factors (expression of *Itgb6* and *RegIII*γ), ultimately protecting the recipient from the lethal consequences of systemic GVHD. In contrast, application of RIG-I agonists 1 day after allo-HSCT did not result in protection and even decreased expression of *Lysozyme P* and *Lgr5*. As we also noticed reduced expression of *Lysozyme P* and *Lgr5* in the gut after TBI and allo-HSCT, we speculated that this lack of therapeutic efficacy of 3pRNA could at least in part be explained by the loss of targetable IECs after pretransplant conditioning. We elucidated the temporal requirements for effective IFN-I–dependent signaling: IFNAR needed to be activated at the time of tissue damage, because early blockade of IFNAR before allo-HSCT but not late blockade after allo-HSCT totally abolished the protective effect of 3pRNA. An earlier study has reported reduction of GVHD if recombinant IFN-a was applied 1 day before allo-HSCT (32). Emphasizing the nonredundant role of the RIG-I/MAVS/IFN-I pathway in epithelial protection, RIG-I ligand–mediated protection was independent of IL-22, a cytokine that enhanced intestinal barrier integrity during allo-HSCT via protection of the ISC compartment (23, 35).

Mechanistically, IFN-I (both RIG-I–/STING-induced and recombinant IFN-β) triggered growth of primary intestinal crypt cultures, an effect that was abrogated by blocking IFNAR. Growth of these epithelial mini-guts relies on sufficient expansion of Lgr5^+^ ISCs that eventually give rise to transient amplifying cells and, ultimately, to mature IECs. Here, we found that organoid formation capacity and production of Paneth cell–derived signals (*Lysozyme P*) were both reduced in *Mavs*^−/−^ allo-HSCT recipients compared to *Mavs*^+/+^ allo-HSCT recipients. In contrast, we could not detect any differences in organoid formation or Paneth cell numbers between *Mavs*^+/+^ and *Mavs*^−/−^ mice in the steady state, suggesting that MAVS and IFN-I might exert their protective functions during acute damage by activation of the ISC compartment. Along these lines, *Sting^gt/gt^* and *Ifnar1*^−/−^ mice also showed defects in organoid formation.

Given that Paneth cells constitute the ISC niche and produce factors that are critical for homeostasis of Lgr5^+^ ISCs and self-renewal in the small intestine (27, 36) including WNT, EGF (epidermal growth factor), and Notch ligands, accurately timed RIG-I– or STING-induced IFN-I signaling could modulate the production of these Paneth cell–derived signals during acute tissue damage. We observed that RIG-I ligands protected Paneth cells in allo-HSCT mouse recipients and enhanced expression of *Lysozyme P* and *Lgr5*. Moreover, RIG-I– and STING-induced IFN-I enhanced the production of *RegIII*γ that could contribute to limiting intestinal tissue damage by sustaining a protective shield against bacterial colonization and translocation (21, 37). Finally, we found that administration of 3pRNA before allo-HSCT allowed retrieval of more organoids from the small intestine of treated recipients compared to untreated control recipients and required the RIG-I adaptor MAVS to induce epithelial regeneration. Engagement of RIG-I in vivo thus augmented ISC function and epithelial regeneration during allo-HSCT. Given that expression of RIG-I, MAVS, and STING has previously been identified in Lgr5^+^ ISCs in a proteomic screen (38), future studies will clarify whether endogenous RIG-I and STING ligands and IFN-I enhance organoid growth by directly acting on Lgr5^+^ ISCs. Alternatively, Bmi^+^ ISCs, considered to be injury-inducible cells with full potential for epithelial regeneration shortly after irradiation damage (39), could be targets for RIG-I/MAVS–, STING–, or IFN-I– dependent signals.

Under conditions of chronic viral challenge and chronic IFN-I signaling, myeloid cells are the main target of IFN-I signals, controlling epithelial barrier integrity through secretion of apolipoproteins L9a/b (10). In addition, natural killer cells (both donor or recipient) reduce inflammation after irradiation-induced gut epithelial barrier loss and GVHD in several mouse models (40) and are activated by IFN-I after 3pRNA injection (22). Non-IEC IFN-I targets could contribute to the 3pRNA-induced protection against gut barrier loss and GVHD. 3pRNA increased expression of apolipoproteins L9a/b in the small intestine of irradiated WT mice, an effect that was entirely dependent on IFN-I signaling. In contrast, weight loss during GVHD in *Ifnar1^fl/fl^ CD11cCre* mice was higher, but reduction of GVHD-associated weight loss by RIG-I activation was not affected. This suggested that although IFN-I signaling through DCs appears to be important for limiting tissue damage under certain conditions, protection from tissue injury in GVHD via RIG-I activation is not mediated by IFN-I signaling in DCs.

Our data suggested that endogenous RIG-I/MAVS and STING signaling resulted in protective IFN-I signaling to maintain epithelial barrier integrity, specifically in the context of tissue damage induced by TBI, chemotherapy, and GVHD. In this respect, identifying endogenous ligands that engage these pathways and mediate protection is of particular interest.

There are a number of limitations to our study. First, the links among the effects of IFN-I signaling on IECs, protection of gut epithelial barrier function, and reduction of GVHD are still correlative, and causal relationships remain to be formally proven. More detailed, large-scale experiments with BM chimeras and inducible, epithelia- or mesenchyme-specific IFNAR^−/−^ mouse recipients are needed. Second, testing the role of RIG-I/MAVS, STING, and IFN-I in additional minor mismatch models of allo-HSCT may allow more general conclusions to be drawn about the relevance of these pathways in allo-HSCT. Similarly, exploring the role of RIG-I/MAVS, STING, and IFN-I activation in additional models of chemotherapy-induced tissue injury might extend our findings that are currently limited to doxorubicin treatment and the analysis of the RIG-I/MAVS pathway. Third, although RIG-I has been shown to be down-regulated in the intestinal epithelium of patients with Crohn's disease (41), the role of cytosolic nucleic acid sensors in human allo-HSCT and GVHD remains unclear. Intestinal gene expression analysis in patients with varying grades of GVHD may provide insights into the role of cytosolic nucleic acid sensors and open the way for prospective studies using RIG-I agonists, which are currently being developed for clinical application. Finally, given that the cytosolic DNA receptor cGAS has been shown to bind to a variety of DNA molecules including IFN-stimulatory DNA (4), we can only speculate that cGAS could be the main sensor upstream of STING to induce the protective function of dsDNA, but this requires further evaluation.

In summary, we have shown that correctly timed therapeutic activation of RIG-I or STING may offer a strategy to reduce gut epithelial barrier dysfunction and promote epithelial integrity during acute tissue damage caused by chemotherapy or TBI and thus help to prevent the development of GVHD.

## Materials and Methods

### Study design

The goal of this study was to evaluate the impact of RIG-I/MAVS and STING signaling on gut integrity during acute tissue injury and GVHD in mice. To assess this, acute tissue damage was induced by TBI, cytotoxic chemotherapy, and mouse models of allo-HSCT. GVHD intensity was quantified using survival, weight loss, histopathology, and immunohistochemistry. Intestinal barrier function was analyzed using FITC-dextran translocation, expression of antimicrobial peptides, and neutrophil influx into the lamina propria. Bacteremia was measured in the serum by counting CFU. Organoid cultures of mouse small intestinal crypts were used as an indicator for epithelial regeneration. Damage-associated DNA release was quantified using total DNA isolated from mouse plasma. qPCR was performed for gene expression analysis of IFN signaling, antimicrobial peptide production, and small intestine stem or Paneth cell marker expression. 16S rRNA sequencing was performed to detect potential differences in the intestinal bacterial composition of WT, *Mavs*^−/−^, or *Sting^gt/gt^* mice. For animal studies, sample sizes were chosen according to the power of the statistical test of each experiment. For all studies, animal numbers are depicted in the figures, and the number of independent experiments is listed in the figure legends. WT and genetically modified mice were randomized into experimental groups and randomly assigned to different cages. Experienced GVHD pathologists performed histopathological scoring of intestinal damage after allo-HSCT in a blinded fashion. All mouse antibodies used in this study were validated with flow cytometry by the supplier, either eBioscience, BD Biosciences, or BioLegend. Mouse antibodies and their clones are listed in table S1 (clone number, 1DegreeBio, reference ID). Cell lines were tested for mycoplasma at frequent intervals. We did not exclude outliers in any experiment. For all results, statistical tests are described in the figure legends.

### Mice

*Mavs*^−/−^ (C57BL/6) were provided by the late J. Tschopp. *Rig-I*^−/−^ mice (129/sv) were provided by Z.-G. Wang (34). *Ifnar1^fl/fl^* × CD11c-Cre mice were provided by U. Kalinke (Hannover, Germany). *Sting^gt/gt^* mice were from the Jackson Laboratory. We used cohoused mice (*Sting^gt/gt^*) or littermates derived from heterozygous breeding pairs as indicated in the results or figure legends.

### BM transplantation

Allogeneic BM transplantation was performed as previously described (42). Recipients were given 5 × 10^6^ T cell–depleted BM cells directly after lethal TBI. T cell depletion of BM cells was performed as previously described (43). T cell doses (MACS enrichment, Miltenyi) varied depending on the transplant model.

### In vivo permeability assay

FITC-dextran assay was performed as previously described (35). FITC-dextran (#FD4-1G, Sigma) was administered by oral gavage at a concentration of 50 mg/ml in water (750 mg/kg). Four and a half hours later, plasma was collected and analyzed on a plate reader.

### In vivo analysis of neutrophil infiltration

Mice were lethally irradiated or treated with doxorubicin. On day 3, lamina propria leukocytes were isolated and neutrophils were analyzed by flow cytometry as previously described (44).

### Analysis of T cell proliferation in vivo

In vivo T cell analysis was performed as previously described (45). Car-boxyluorescein diacetate succinimidyl ester (15 × 10^6^) (eBioscience)–stained T cells were transplanted into lethally irradiated allogeneic recipients as described above.

### Crypt isolation

Isolation of intestinal epithelial crypts was performed as previously described (26). Briefly, small intestines were opened longitudinally, washed, and incubated in 10 mM EDTA for 25 min (4°C). Supernatant containing crypts was collected.

### Organoid culture

Two hundred fifty crypts per well were suspended in growth factor–reduced Matrigel (Corning) (33% ENR medium; 66% Matrigel) at 4°C and plated in 30-μl drops, each containing about 250 crypts. Five hundred microliters of ENR-medium [Advanced DMEM/F-12 (Life Technologies), 2 mM l-glutamine (Sigma), 10 mM Hepes (Life Technologies), penicillin (100 U/ml)/streptomycin (100 μg/ml) (Life Technologies), 1.25 mM N-acetyl cysteine (Sigma), 1 × B27 supplement (Life Technologies), 1 × N2 supplement (Life Technologies), mEGF (50 ng/ml) (PeproTech), recombinant murine Noggin (100 ng/ml) (PeproTech), 5% human R-spondin-1-conditioned medium of hR-spondin-1–transfected human embryonic kidney (HEK) 293T cells] was added to crypt cultures. Medium was replaced every 2 to 3 days. 3pRNA and IFN-stimulatory DNA were complexed with Lipofectamine 2000 (Invitrogen). Recombinant murine IFN-β [PBL (12400-1)] and/or IFNAR1 antibody/IgG1 isotype control were added with medium change.

### Quantitative PCR

Total RNA was isolated and transcribed using standard methods and kits according to the manufacturer's protocols (RNeasy Mini Kit, Qiagen; SuperScript III Reverse Transcriptase, Invitrogen). The qPCR Core Kit for SYBR Green I (Eurogentec) and the LightCycler 480 II (Roche) Real-Time PCR System were used as indicated by the manufacturer. The relative transcript level of each gene was calculated according to the 2^−^*^C^*^t^, for unnormalized genes, and the 2^−ΔΔ^*^C^*^t^ method, for genes normalized to β-actin.

### Assessment of epithelial regeneration in intestinal organoid cultures

To determine organoid size and morphology, bright-field microscopy images were taken using a Zeiss Axiovision Observer microscope with a 5× objective lens. Two-dimensional area and perimeter were analyzed using border perimeter tracing of organoids of each well using ImageJ software.

### Reagents

Double-stranded in vitro–transcribed 3pRNA (sense, 5′-UCAAACAGUCCUCGCAUGCCUAUAGUGAGUCG-3′) was generated as described (22).

### Treatments before allo-HSCT

*Mice* were treated at indicated time points with 3pRNA or IFN-stimulatory DNA (25 μg if not indicated otherwise). 3pRNA or IFN-stimulatory DNA was complexed in 3.5 μl of in vivo-jetPEI (Polyplus) and injected intravenously. In some experiments, mice were treated intraperitoneally with 500 μg of IFNAR1-blocking antibody/IgG1 control.

### Statistics

Animal numbers per group (*n*) are depicted in the figure legends. We never used technical replicates. GraphPad Prism version 6 was used for statistical analysis. Survival was analyzed using the log-rank test. Differences between means of experimental groups were analyzed using two-tailed unpaired *t* test or ordinary one-way ANOVA. We used ordinary one-way ANOVA for multiple comparisons and always performed Dunnett's test for multiple test corrections. Applied statistical tests are indicated in the figure legends. Significance was set at *P < 0.05, **P < 0.01, and ***P < 0.001. Data are means ± SEM.

## Supplementary Material

Supplemental Table S1. Antibodies.
Fig. S1. Endogenous RIG-I/MAVS signaling reduces intestinal tissue damage caused by conditioning therapy and attenuates GVHD.
Fig. S2. Donor-derived T cells show enhanced alloreactivity in *Mavs*^−/−^ allo-HSCT recipients.
Fig. S3. RIG-I ligands have to be applied before or during allo-HSCT to exert their protective effects and do not affect GVL.
Fig. S4. RIG-I–induced treatment effects are mediated by IFN-Is.
Fig. S5. RIG-I–induced IFN-Is enhance epithelial regeneration through stimulation of the ISC compartment.
Fig. S6. MAVS-deficient mice do not display an inherent defect in organoid formation or in the number of Paneth cells.
Fig. S7. TBI and IFN-stimulatory DNA induce a systemic IFN-I response, and feces-derived RNA triggers a RIG-I–dependent IFN-I response in IECs.
