# Supplementary material for: RIG-I/MAVS and STING signaling promote gut integrity during irradiation- and immune-mediated tissue injury
Source: Sci Transl Med. Author manuscript; Available in PMC 2017 Sep 19. (PMC5604790; doi:10.1126/scitranslmed.aag2513)
Supplement: Supplemental — Table S1. Antibodies. Fig. S1. Endogenous RIG-I/MAVS signaling reduces intestinal tissue damage caused by conditioning therapy and attenuates GVHD. Fig. S2. Donor-derived T cells show enhanced alloreactivity in Mavs−/− allo-HSCT recipients. Fig. S3. RIG-I ligands have to be applied before or during allo-HSCT to exert their protective effects and do not affect GVL. Fig. S4. RIG-I–induced treatment effects are mediated by IFN-Is. Fig. S5. RIG-I–induced IFN-Is enhance epithelial regeneration through stimulation of the ISC compartment. Fig. S6. MAVS-deficient mice do not display an inherent defect in organoid formation or in the number of Paneth cells. Fig. S7. TBI and IFN-stimulatory DNA induce a systemic IFN-I response, and feces-derived RNA triggers a RIG-I–dependent IFN-I response in IECs. [file NIHMS896799-supplement-Supplemental.pdf]

Science Translational Medicine
AAAS

www.sciencetranslationalmedicine.org/cgi/content/full/9/386/eaag2513/DC1

Supplementary Materials for

RIG-I/MAVS and STING signaling promote gut integrity during irradiation- and immune-mediated tissue injury

Julius C. Fischer, Michael Bscheider, Gabriel Eisenkolb, Chia-Ching Lin, Alexander Wintges, Vera Otten, Caroline A. Lindemans, Simon Heidegger, Martina Rudelius, Sébastien Monette, Kori A. Porosnicu Rodriguez, Marco Calafiore, Sophie Liebermann, Chen Liu, Stefan Lienenklaus, Siegfried Weiss, Ulrich Kalinke, Jürgen Ruland, Christian Peschel, Yusuke Shono, Melissa Docampo, Enrico Velardi, Robert R. Jenq, Alan M. Hanash, Jarrod A. Dudakov, Tobias Haas, Marcel R. M. van den Brink,* Hendrik Poeck*

*Corresponding author. Email: hendrik.poeck@tum.de (H.P.); m-van-den-brink@ski.mskcc.org (M.R.M.v.d.B.)

Published 19 April 2017, *Sci. Transl. Med.* **9**, eaag2513 (2017)
DOI: 10.1126/scitranslmed.aag2513

This PDF file includes:

Materials and Methods
Table S1. Antibodies.
Fig. S1. Endogenous RIG-I/MAVS signaling reduces intestinal tissue damage caused by conditioning therapy and attenuates GVHD.
Fig. S2. Donor-derived T cells show enhanced alloreactivity in *Mavs*$^{-/-}$ allo-HSCT recipients.
Fig. S3. RIG-I ligands have to be applied before or during allo-HSCT to exert their protective effects and do not affect GVL.
Fig. S4. RIG-I–induced treatment effects are mediated by IFN-Is.
Fig. S5. RIG-I–induced IFN-Is enhance epithelial regeneration through stimulation of the ISC compartment.
Fig. S6. MAVS-deficient mice do not display an inherent defect in organoid formation or in the number of Paneth cells.
Fig. S7. TBI and IFN-stimulatory DNA induce a systemic IFN-I response, and feces-derived RNA triggers a RIG-I–dependent IFN-I response in IECs.

Materials and Methods

Mice

C57BL/6 (H-2k^b, Thy-1.2), BALB/c (H-2k^d, Thy-1.2) were purchased from Janvier Labs (France). *Mavs*$^{-/-}$ (C57BL/6) were provided by the late J. Tschopp. *Ifnar1*$^{-/-}$ (C57BL/6) mice were provided by Joseph C. Sun (MSKCC), *Il-22*$^{-/-}$ (Balb/c) mice were provided by Genentech. *Rig-I*$^{-/-}$ mice (129/sv) were provided by Zhu-gang Wang (State Key Laboratory of Medical Genomics, Shanghai Jiao Tong University School of Medicine, Shanghai 200025, P.R.China) (*34*). *Ifn-β*$^{\Delta\beta\text{-}luc}$ mice used for in vivo imaging were backcrossed to C57BL/6 albino background (*42*). Floxed Ifnar1 mice (C57BL/6) crossed with CD11c-Cre mice (C57BL/6) were provided by U. Kalinke (Twincore, Hannover, Germany). *Sting*$^{gt/gt}$ mice were from Jackson (Stock number 017537). Mice were used between 6 and 12 weeks of age at the onset of experiments and were maintained in specific pathogen free conditions. We used littermates derived from heterozygous breeding pairs (*Mavs*$^{-/-}$, *Mavs*$^{+/+}$; Ifnar1$^{fl/fl}$ CD11c-Cre^{+}, Ifnar1$^{fl/fl}$ CD11c-Cre^{-} ; *Rig-I*$^{-/-}$, *Rig-I*$^{+/-}$) or cohoused mice as indicated in the results or figure legends. Animal studies were approved by the local regulatory agencies (Regierung von Oberbayern, Munich, and Landesamt für Verbraucherschutz und Lebensmittelsicherheit (LAVES), Oldenburg, Germany) and by the Memorial Sloan-Kettering Cancer Center (MSKCC) Institutional Animal Care and Use Committee (IACUC).

Bone marrow transplantation model

Allogeneic bone marrow transplants were performed as previously described (*43*). Briefly, recipients were given 5x10^6 BM cells directly after lethal total body irradiation (TBI) with 2x4.5Gy (BALB/c), 2x5.5Gy (C57BL/6) or 2x5Gy (129/sv). T cell doses (CD4/CD8 or CD5 MACS enrichment, Miltenyi) varied depending on the transplant model: Donor C57BL/6 into recipient BALB/c (0.5x10^6 or 1x10^6 when indicated), donor BALB/c into recipient C57BL/6 (2x10^6), donor C57BL/6 into recipient 129/sv (1x10^6), donor B10.BR into C57BL/6 (1x10^6). We used T cell depleted BM in all allo-HSCT experiments with BM only controls. T cell depletion of BM cells was performed as previously described (*44*)

Generation of chimeric recipients with *Mavs* or *Ifnar1* deficiency of hematopoietic or non-hematopoietic tissues

WT, *Mavs*$^{-/-}$ and *Ifnar1*$^{-/-}$ recipients (C57BL/6J) were injected as syngeneic bone marrow transplantation (BMT) with 5 x 10^6 WT or *Mavs*$^{-/-}$ or *Ifnar1*$^{-/-}$ BM cells (C57Bl/6) intravenously directly after TBI with 2x5.5Gy. Between 45 and 90 days after first syngeneic BMT, allogeneic HSCT (donor BALB/c into recipient C57Bl/6: T cell dose 2x10^6, TBI 2x5.5Gy; donor B10.BR into recipient C57BL/6: T cell dose 1x10^6, TBI 2x4.5 Gy) was performed.

In vivo permeability assay (FITC-dextran)

FITC-dextran Assay was performed as previously described (*35*). Mice were kept without food and water for 8 hours and then FITC-dextran (#FD4-1G, Sigma) was administered by oral gavage at a concentration of 50 mg/ml in water (750mg/kg). 4.5 hours later, plasma was collected from peripheral blood (8800rcf, 10min), then mixed 1:1 with PBS and

analyzed on a plate reader at an excitation wavelength of 485 nm and an emission wavelength of 535 nm.

Determination of bacteremia

To determine bacteremia, peripheral blood was collected and centrifuged at 400g for 5 min, supernatant (blood plasma) was collected, plated and incubated at 37°C under anaerobic conditions using Columbia Agar plates. After 48h CFUs were counted and bacteremia was quantified in CFUs per ml blood plasma.

Isolation of Lamina Propria Leukocytes and intestinal epithelial cells (IEC) from the small intestine

Isolation was performed as previously described (*43*). Briefly, Peyer's patches were excised from ileum (defined as distal 1/3 of small intestine) and ileums were flushed with cold PBS and cut into 2 cm pieces. Longitudinally opened intestines were washed and incubated with HBSS solution containing 2mM EDTA, 10 mM HEPES, 10% FCS (Hyclone), 1% Penicillin-Streptomycin, 1 % L-Glutamine and 1 mM DTT (all Sigma-Aldrich). After incubation on a shaker (225 rpm) at 37°C for 2 x 15 min, tissues were washed and filtered through a 100 μm strainer (BD 352360). The flow-through were centrifuged for 5 min at 1,500 r.p.m and the remaining pellet was lysed in TRIzol (Ambion) for subsequent RNA extraction. Next, intestines were incubated for 45min in $PBS^{+Ca/+Mg}$ supplemented with FCS (10%), Collagenase II (200 U/ml; Worthington), and DNase I (0.05 mg/ml; Roche) on a shaker at 37°C. Lamina Propria Leukocytes (LPL) in suspension were then purified on a 40/80% Percoll gradient (Biochrom).

In vivo analysis of neutrophil infiltration

Phenotypical analysis of neutrophils was performed as previously described (*45*). For assessment of neutrophil infiltration after TBI or doxorubicin treatment, 6-12 weeks old mice were irradiated with 9Gy (Balb/c) or 11Gy (C57BL/6) or treated with doxorubicin injected intraperitoneally (i.p.) (7.5mg/KG body weight, unless indicated otherwise). On day 3 after intervention mice were sacrificed, Lamina Propria Leukocytes were isolated, counted and neutrophils within the LPLs were analyzed by flow cytometry and normalized to the absolute number of averagely isolated cells ($1x10^6$)

Flow cytometry

Cell suspensions were stained in PBS with 3% FCS. Fluorochrome-coupled antibodies were purchased from eBioscience or BioLegend and are listed Table 1. For intracellular cytokine staining (ICS), T cells were activated with 80 nM Phorbol-12-myristat-13- acetat (PMA; Sigma), 1μM ionomycin (Merck Millipor) and Brefeldin A for 4 hours. For ICS, the Foxp3 Transcription Factor Fixation/Permeabilization Kit (eBioscience) was used according to manufacturer's instructions. Data were acquired on a FACS Canto II (BD Biosciences) and analyzed using FlowJo software (TreeStar).

Analysis of T cell proliferation *in vivo*

In vivo T cell analysis was performed as previously described (*46*). T cell and BM preparation was performed as described above. T cells were stained with 3.5 μM carboxyfluorescein diacetate succinimidyl ester (CFSE, eBioscience) for 12 minutes at

37°C, washed and counted. 15 x 10^6 stained cells were transplanted into lethally irradiated allogeneic recipients as described above. Spleens were harvested on day 3 and analyzed with FACS.

Crypt isolation

Isolation of intestinal epithelial crypts was performed as previously described (*26*). Briefly, after harvesting small intestines, the organs were opened longitudinally and washed. Small intestine was incubated in 10 mM ethylenediamine-tetraacetatic acid (EDTA) for 25 min (4°C) to dissociate the crypts. The supernatant containing crypts was collected.

Organoid culture

250 crypts per well were suspended in liquefied growth factor reduced Matrigel (Corning) (33% ENR-medium; 66% growth factor reduced Matrigel) at 4°C. Then, they were plated in delta-surface Nunc 24-well plates in 30 µL drops, each containing approximately 250 crypts. After the Matrigel drops polymerized, 500ul complete crypt culture medium was added to small intestine crypt cultures (ENR-medium: advanced DMEM/F12 (Life technologies), 2 mM L-glutamine (Sigma), 10 mM HEPES (Life technologies), 100 U/ml penicillin/100 µg/ml streptomycin (Life technologies), 1.25 mM N-acetyl cysteine (Sigma), 1x B27 supplement (Life technologies), 1x N2 supplement (Life technologies), 50 ng/ml mEGF (Peprotech), 100ng/ml rec. mNoggin (Peprotech), 5% human R-spondin-1 conditioned medium of hR-spondin-1-transfected HEK 293T cells). Together with the crypt culture medium, 2µg/ml of 3pRNA or 2µg/ml of ISD complexed with Lipofectamine 2000 (Invitrogen) or recombinant murine (rm) IFN-β (20U/ml; PBL (12400-1)) was added. All plates were incubated at 37 °C/5% CO2 and medium was replaced every 2-3 days. IFN-β was added again with every medium change. For IFNαR1 blockade, 10ug/ml of antibody were added to the matrigel before polymerization and with every medium change (MAR1-5A3 anti-mIFNalphaR1 antibody or MOPC-21 Mouse IgG1 as isotype control (BioXCell)).

Histopathologic analysis

Intestines were harvested 8 days after allo-HSCT or 72 hours after TBI for histopathologic assessment of intestinal tissue injury. Samples were formalin-preserved, paraffin-embedded, sectioned, and stained with hematoxylin and eosin (H&E). For evaluation of intestinal GVHD after allo-HSCT, blinded scoring was performed by experienced pathologists (C.L. or M.R.) as previously described (*47*). For evidence of intestinal tissue damage after TBI, tissues were examined by four established criteria in a blinded fashion by a pathologist (S.M.): crypt apoptosis (% of crypt containing at least 1 apoptotic cell), crypt abscesses (Absent (0), Present (1)), granulocytic infiltrates (Absent (0), minimal (1), mild (2), moderate (3), marked (4)) and villus atrophy (absent (0), minimal (1), mild (2), moderate (3), marked (4)). Each mouse was given an individual cumulative score (histopathology score) based on the above criteria.

Immunohistochemistry

Intestines of mice 8 days after allo-HSCT were harvested, formalin-fixed, paraffin embedded. The immunohistochemical detection of Lysozyme was performed using Discovery XT processor (Ventana Medical Systems). The tissue sections were

deparaffinized with EZPrep buffer (Ventana Medical Systems), antigen retrieval was performed with CC1 buffer (Ventana Medical Systems) and sections were blocked for 30 minutes with Background Buster solution (Innovex). Slides were incubated with anti-Lysozyme antibodies (DAKO; cat# A099; 2ug/ml) for 5h, followed by 60 minutes incubation with biotinylated goat anti-rabbit IgG (Vector labs, cat#PK6101) at 1:200 dilution. The detection was performed with DAB detection kit (Ventana Medical Systems) according to manufacturer´s instruction. Slides were counterstained with hematoxylin (Ventana Medical Systems) and coverslipped with Permount (Fisher Scientific). To quantify Lysozyme^{+} Paneth cells, the number of positive cells per crypt was evaluated over a 5000 µm length of intestinal mucosa. Lysozyme^{+} Paneth cells are depicted as mean Paneth cell number / crypt. Quantification was performed in a blinded fashion by S.M.

Detection of bioluminescence and in vivo imaging

Ifn-β$^{\Delta\beta\text{-luc}}$ mice were injected i.v. with 100 µl luciferin (30 mg/ml in PBS)/20 g mouse weight and anesthesized using isoflurane. Within 10 min after luciferin injection, mice or isolated organs were analyzed with an in vivo imaging instrument (IVIS 200; PerkinElmer). The acquired images were analyzed using Living Image 4.4. software.

Quantitative PCR

RNA was isolated from cells lysed in TRIzol (ambion) or from whole tissue homogenates. Tissue homogenates were prepared as follows: 1 cm large or small intestine was flushed and longitudinally opened pieces were frozen in 500ul TRIzol reagent using liquid nitrogen. After thawing, samples were supplemented with stainless stell beads 5mm (Qiagen) and homogenized using a Tissuelyser II (Qiagen) 1 min with 30Hz (1800 oscillations/minute). Total RNA was isolated and transcribed using standard methods and kits according to manufacturer´s protocols (RNeasy Mini Kit, Qiagen; SuperScript III Reverse Transcriptase, invitrogen). The specific primer pairs were as follows: mRegIII*γ* fwd TTCCTGTCCTCCATGATCAAAA, rev CATCCACCTCTGTTGGGTTCA; mActin fwd CACACCCGCCACCAGTTCG, rev CACCATCACACCCTGGTGC; mLgr5 fwd ACCCGCCAGTCTCCTACATC rev GCATCTAGGCGCAGGGATTG; mLysozymeP fwd CAG GCCAAGGTCTACAATCG, rev TTGATCCCACAGGCATTCTT.; mItgb6 fwd ATTGTCATTCCCAATGATGG, rev CATAGTTCTCATACAGATGGAC. The qPCR Core kit for SYBR Green I (Eurogentec) and a LightCycler 480 II (Roche) Real-Time PCR System were used as indicated by the manufacturer. The relative transcript level of each gene was calculated according to the 2−Ct, for unnormalized genes, and the 2−ΔΔCt method, for the genes normalized to *β-Actin*. Alternatively, the following Taqman Expression Assay IDs were used: BETA-ACTIN Mm01205647_g1; IFNB1 Mm00439552_s1; REG3G Mm00441127_m1;

Measurement of cytokines

TNF and IL-6 were analyzed using the Cytometric Bead Array Enhanced Sensitivity Flex Set System (BD) according to manufacturer’s instructions. IFNα and IFNβ were analyzed by ELISA (PBL Assay Science) according to manufacturer’s instructions.

Assessment of epithelial regeneration in intestinal organoid cultures

To determine the effect of 3pRNA / interferon stimulatory DNA / rmIFN-β on organoid size and morphology, bright-field microscopy images were taken using a Zeiss Axiovision Observer microscope with a 5x objective lens after 5 or 7 days in culture. 2D area and perimeter were analyzed using border perimeter tracing of organoids found in four representative fields of each well using Image J software. For assessment of gene expression by quantitative (q) PCR, organoids were subjected to RNA extraction 24 hours after culture using Trizol reagent (Invitrogen) according to manufacturer's protocol. Isolated RNA was reverse-transcribed using the Quantitect Reverse Transcription Kit (Qiagen). Gene expression was assessed by quantitative real-time PCR using Taqman Expression Assay pre-designed probes (Applied Biosystems). Signals were normalized to *β-Actin*. mRNA expression. Normalized values were used to calculate relative expression by ΔΔCt analysis or absolute expression by ΔCt. Taqman IDs are depicted below (qPCR).

Reagents

OptiMEM reduced-serum medium was from Invitrogen. Double-stranded in vitro-transcribed 3pRNA (sense, 5′- UCA AAC AGU CCU CGC AUG CCU AUA GUG AGU CG -3′) was generated as described (*22*). Synthetic dsRNA with the same sequence but lacking the 5′-triphosphate (synRNA) was purchased from Eurofins (Ebersberg, Germany). Interferon stimulatory DNA was purchased from Invivogen.

Drug treatment

Mice were treated on indicated time points with 3pRNA or interferon stimulatory DNA (25 µg if not indicated otherwise). 3pRNA / interferon stimulatory DNA was complexed in 3.5 µl in vivo-jetPEI (Polyplus) and injected intravenously. In some experiments mice were treated i.p. with 500ug IFNaR1 blocking antibody (Clone: MAR1-5A3, BioXCell, West Lebanon, NH) or IgG1 Isotype control (Clone: MOPC-21, BioXCell, West Lebanon, NH) as indicated.

16S RNA gene Sequencing

Stool specimens were stored at -80°C. DNA was purified using a phenol-chloroform extraction technique with mechanical disruption (bead-beating) based on a previously described protocol (*48*) and analyzed using the Illumina MiSeq platform to sequence the V4-V5 region of the 16S rRNA gene. Sequence data were compiled and processed using mothur version 1.34(*49*), screened and filtered for quality (*50*), then classified to the species level (*51*) using a modified form of the Greengenes reference database (*52*), screened and filtered for quality (*50*), then classified to the species level (*51*) using a modified form of the Greengenes reference database (*52*).

Quantification of Plasma DNA levels

Mouse plasma was collected from peripheral blood (8800rcf, 10min). Plasma samples of 3-4 mice were combined to a final volume of 400-500µl and DNA extracted using the QIAamp Circulating Nucleic Acids Kit (Qiagen). dsDNA was quantified using a Qubit 2.0 Fluorometer with the Qubit dsDNA HS Assay Kit (Thermo Fisher Scientific).

GVT model and bioluminescence imaging
A20-TGL (H-2^d), a BALB/c B-cell lymphoma, were generated as described previously (*53*). A20-TGL tumor cells were inoculated via separate intravenous injection on the day of allo-BMT (*54*). To visualize and quantify tumor burden, A20-TGL inoculated mice were administered D-luciferin (Goldbio), anesthetized, and imaged using in vivo bioluminescence imaging systems (Caliper Life Sciences)

Cell lines, culture and RNA transfection, feces RNA isolation
Mode-K cells were purchased from Dominique Kaiserlian (French Institute of Health and Medical Research, Unit of Immunity Infection Vaccination, France) and cultured as previously described (*31*). Cell lines were tested as mycoplasma negative. Where indicated, MODE-K cells were transfected with mouse RIG-I siRNA (100µM, Eurofins Genomics,) or control siRNA (Qiagen) using Lipofectamine 2000 (Life Technologies) according to manufacturer's instructions. After 48 h, cells were transfected with 3pRNA (0.8µg/mL) or mouse feces-derived RNA complexed to Lipofectamine 2000. Supernatants were collected and RNA was extracted 18h after transfection followed by IFN-β measurement with ELISA (PBL Assay Science) or by assessment of IFN-β mRNA by qPCR. Mouse feces from healthy WT mice was diluted (RNAprotect Reagent, Quiagen) and homogenized with Glass beads (Sigma) and a Tissuelyser II (Qiagen). After centrifugation, supernatant was subtracted and total feces RNA was isolated using standard methods and kits according to manufacterers' protocols.

Gene Expression Profiling Analysis
For gene expression profiling analysis, (i) Balb/c mice were solely irradiated (9Gy) (n=3), (ii) pretreated with 3pRNA prior (d-1) to irradiation (n=3) or (iii) pre-treated with 3pRNA (d-1) + α-IFNaR1 blocking antibody (d-2) prior to irradiation (n=3). RNA from small intestines was isolated 12 h after irradiation and used for RNA sequencing. Poly(A) RNA sequencing was performed with three biological replicates for each group and analyzed with an Illumina HiSeq2500 platform. The heatmap depicted in Fig. S4D shows all genes listed in the interferome database (*55*) that show significantly changed gene expression of 3pRNA pretreated and irradiated mice compared to both the other groups simultaneously.

Data Analyis
The output data (FASTQ files) were mapped to the target genome using the rnaStar aligner that maps reads genomically and resolves reads across splice junctions. We used the 2 pass mapping method in which the reads are mapped twice. The first mapping pass used a list of known annotated junctions from Ensemble. Novel junctions found in the first pass were then added to the known junctions and a second mapping pass was done. After mapping we computed the expression count matrix from the mapped reads using HTSeq (www-huber.embl.de/users/anders/HTSeq) and one of several possible gene model databases. The raw count matrix generated by HTSeq was then processed using the R/Bioconductor package DESeq (www-huber.embl.de/users/anders/DESeq) which was used to both normalize the full dataset and analyze differential expression between sample groups.

A heatmap was generated using the heatmap.2 function from the gplots R package. The data plot was the mean centered normalized log2 expression of the top 100 significant

genes. For simple hierarchical clustering the correlation metric was used (Dij = 1 - cor(Xi,Xj)) with the Pearson correlation on the normalized log2 expression values.

Statistics

Animal numbers per group (n) are depicted in the figure legends. We never used technical replicates. GraphPad Prism version 6 was used for statistical analysis. Survival was analyzed using the Log-rank test. Differences between means of experimental groups were analyzed using two-tailed unpaired t test or ordinary one-way Anova correspondingly to the distribution shape of our observations. We used ordinary one-way Anova for multiple comparisons and always performed Dunnett's test for Multiple-test corrections. Applied statistical tests are indicated in the figure legends. Significance was set at p values < 0.05, $p < 0.01$ and $p < 0.001$ and was then indicated with asterisks (*, ** and ***). Data are presented as mean ± S.E.M.

Table S1. Antibodies.

Target structure	Clone#	1DegreeBio ID
CD11b	M1/70	1DB-001-0001021785
CD11c	N418	1DB-001-0000839554
CD3	17A2	1DB-001-0001110661
CD4	GK1.5	1DB-001-0000263404
CD45.1	A20	1DB-001-0000839250
CD45.2	104	1DB-001-0000839196
CD8a	53-6.7	1DB-001-0000263247
IFNaR1	MAR1-5A3	1DB-001-0000840263
IFN γ	XMG1.2	1DB-001-0001110823
Ly-6G/Ly-6C (Gr-1)	RB6-8C5	1DB-001-0000839101

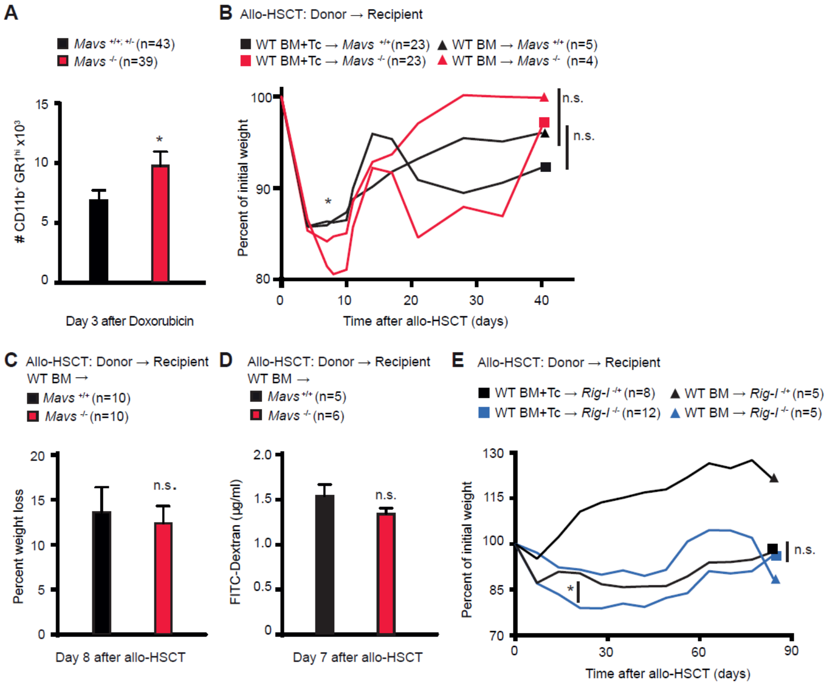

Figure S1

Figure S1. Endogenous RIG-I / MAVS signaling reduces intestinal tissue damage by conditioning therapy and attenuates GVHD. (A) LPL isolated from small intestines of *Mavs*$^{+/+}$/ *Mavs*$^{+/-}$ and *Mavs*$^{-/-}$ mice were analyzed by flow cytometry on day 3 after treatment with doxorubicin (20mg/kg). Pooled data of 3 independent experiments. Animal numbers per group (n) are depicted. **(B)** Weight loss of *Mavs* $^{+/+}$ and *Mavs*$^{-/-}$ animals after TBI + 5x10^{6} BM alone or BM with 2x10^{6} T cells (donor BALB/c into recipient C57BL/6). Pooled data of 4 independent experiments. Animal numbers per group (n) are depicted. **(C)** Weight loss on day 8 after allo-HSCT of *Mavs*$^{+/+}$ and *Mavs*$^{-/-}$ animals after TBI + 5x10^{6} BM alone (donor BALB/c into recipient C57BL/6). Pooled data of (B) and one additional independent experiment. Animal numbers per group (n) are depicted. **(D)** FITC-dextran concentrations in the serum of *Mavs*$^{+/+}$ *and Mavs*$^{-/-}$ recipients on d7 after TBI + 5x10^{6} BM alone (donor BALB/c into recipient C57BL/6). Animal numbers per group (n) are depicted. (**E**) Weight loss of *Rig-I* $^{-/+}$ and *Rig-I* $^{-/-}$ animals after TBI + 5x10^{6} BM alone or BM with 1x10^{6} T cells (donor C57BL/6 into recipient 129/sv). Animal numbers per group (n) are depicted. Experiments were analyzed using two-tailed unpaired t test. Significance was set at p values < 0.05, p < 0.01 and p < 0.001 and was then indicated with asterisks (*, ** and ***). Data are presented as mean ± S.E.M

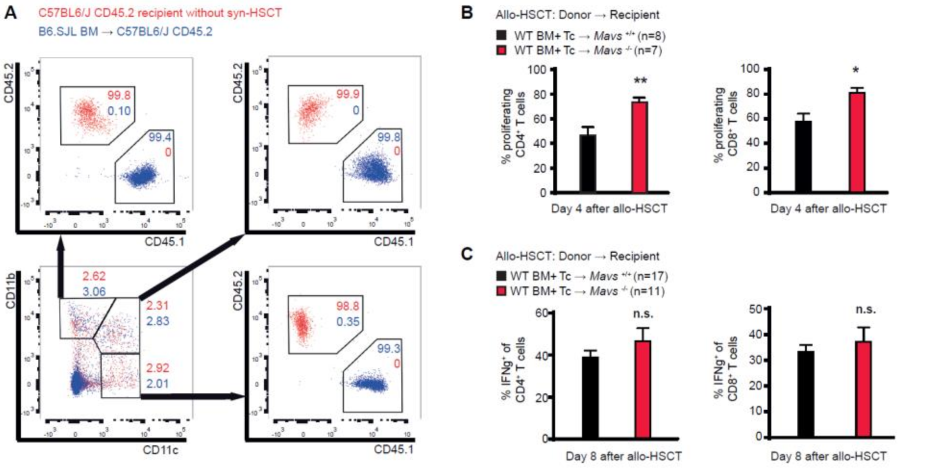

Figure S2

Figure S2. Donor-derived T cells show enhanced alloreactivity in *Mavs*$^{-/-}$ allo-HSCT recipients. (A) CD45.2^{+} C57BL/6 recipients (n=3) received TBI (11Gy) and 5x10^{6} syngeneic CD45.1^{+} BM and were analyzed 43 days after BMT and compared to CD45.2^{+} animals that did not receive TBI and BMT (n=3). Shown is the analysis of small intestine live lamina propria leukocytes (LPL) of one representative animal per group. **(B)** *Mavs*$^{+/+}$ and *Mavs*$^{-/-}$ littermates received TBI + 5x10^{6} BM cells and 15 x10^{6} CFSE labeled T cells (donor BALB/c into recipient C57BL/6). On day 4 after allo-HSCT splenic cells were analyzed by flow cytometry to identify proliferating CFSE labeled donor T-cells. Pooled data of 2 independent experiments. Animal numbers per group (n) are depicted. **(C)** LPL isolated from small intestines of *Mavs*$^{+/+}$ and *Mavs*$^{-/-}$ mice that received TBI + 5x10^{6} BM cells and 2 x10^{6} T cells (donor BALB/c into recipient C57BL/6) were analyzed on day 8 after allo-HSCT by flow cytometry. Pooled data of 2 independent experiments. Animal numbers per group (n) are depicted. All experiments were analyzed using two-tailed unpaired t test. Significance was set at p values < 0.05, p < 0.01 and p < 0.001 and was then indicated with asterisks (*, ** and ***). Data are presented as mean ± S.E.M.

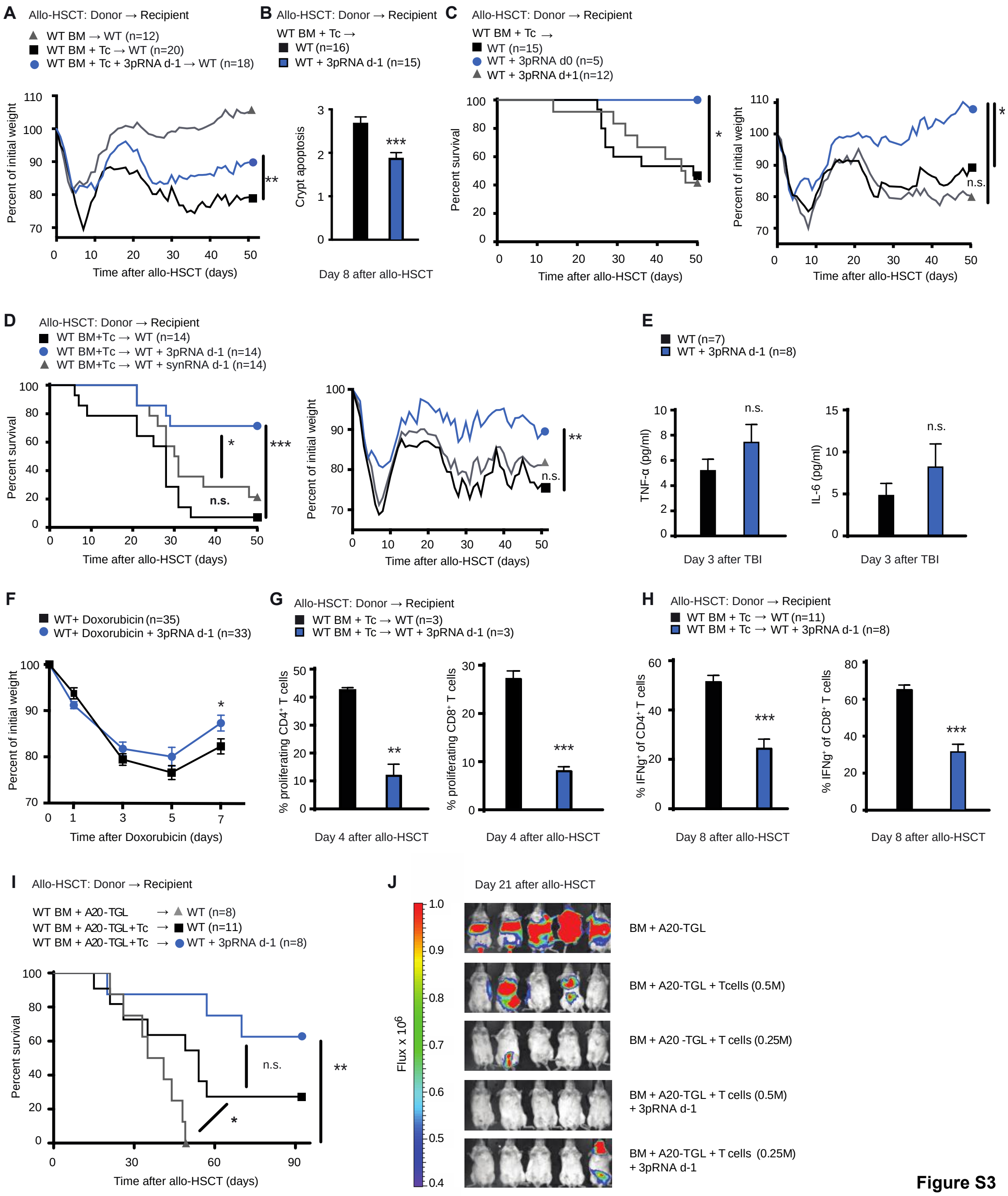

A Allo-HSCT: Donor → Recipient
WT BM → WT (n=12)
WT BM + Tc → WT (n=20)
WT BM + Tc + 3pRNA d-1 → WT (n=18)
Percent of initial weight
Time after allo-HSCT (days)
**
B Allo-HSCT: Donor → Recipient
WT BM + Tc →
WT (n=16)
WT + 3pRNA d-1 (n=15)
Crypt apoptosis
***
Day 8 after allo-HSCT
C Allo-HSCT: Donor → Recipient
WT BM + Tc →
WT (n=15)
WT + 3pRNA d0 (n=5)
WT + 3pRNA d+1(n=12)
Percent survival
Time after allo-HSCT (days)
Percent of initial weight
n.s.
D Allo-HSCT: Donor → Recipient
WT BM+Tc → WT (n=14)
WT BM+Tc → WT + 3pRNA d-1 (n=14)
WT BM+Tc → WT + synRNA d-1 (n=14)
Percent survival
n.s.
Time after allo-HSCT (days)
Percent of initial weight
E
WT (n=7)
WT + 3pRNA d-1 (n=8)
TNF-α (pg/ml)
IL-6 (pg/ml)
Day 3 after TBI
F
WT+ Doxorubicin (n=35)
WT+ Doxorubicin + 3pRNA d-1 (n=33)
Percent of initial weight
Time after Doxorubicin (days)
G Allo-HSCT: Donor → Recipient
WT BM + Tc → WT (n=3)
WT BM + Tc → WT + 3pRNA d-1 (n=3)
% proliferating CD4+ T cells
% proliferating CD8+ T cells
Day 4 after allo-HSCT
H Allo-HSCT: Donor → Recipient
WT BM + Tc → WT (n=11)
WT BM + Tc → WT + 3pRNA d-1 (n=8)
% IFNg+ of CD4+ T cells
% IFNg+ of CD8+ T cells
Day 8 after allo-HSCT
I Allo-HSCT: Donor → Recipient
WT BM + A20-TGL → WT (n=8)
WT BM + A20-TGL+Tc → WT (n=11)
WT BM + A20-TGL+Tc → WT + 3pRNA d-1 (n=8)
Percent survival
Time after allo-HSCT (days)
J
Day 21 after allo-HSCT
Flux x 10^6
BM + A20-TGL
BM + A20-TGL + Tcells (0.5M)
BM + A20 -TGL + T cells (0.25M)
BM + A20-TGL + T cells (0.5M) + 3pRNA d-1
BM + A20-TGL + T cells (0.25M) + 3pRNA d-1

Figure S3

Figure S3. RIG-I ligands have to be applied before or during allo-HSCT to exert their protective effects and do not affect GVL. (**A**) Weight loss of animals after 9Gy TBI + $5x10^6$ BM alone with $1x10^6$T cells (donor C57BL/6 into recipient BALB/c). Indicated mice were either left untreated or treated with 3pRNA on day -1. Pooled data of 4 independent experiments. Animal numbers per group (n) are depicted. (**B**) Histopathological analysis of crypt apoptosis of small intestines from allo-HSCT recipients (donor C57BL/6 into recipient BALB/c). Indicated mice were either left untreated or treated with 3pRNA on day -1. Pooled data of 3 independent experiments. Animal numbers per group (n) are depicted. (**C**) Survival and weight loss of allo-HSCT recipients (donor C57BL/6 into recipient BALB/c). Indicated mice were either left untreated or treated with 3pRNA on day 0 or d+1. Pooled data of 3 independent experiments. Animal numbers per group (n) are depicted. **(D)** Survival and weight loss of allo-HSCT recipients (donor C57BL/6 into recipient BALB/c, 9Gy TBI + $5x10^6$ BM + $1x10^6$T cells). Indicated mice were left untreated or treated with 3pRNA or non-triphosphorylated control RNA (synRNA) on day -1. Pooled data of 2 independent experiments. Animal numbers per group (n) are depicted. **(E)** Measurement of serum cytokines of BALB/c mice on day 3 after TBI (9 Gy) using cytometric bead array (CBA). Indicated mice were left untreated or treated with 3pRNA on d-1. Pooled data of 3 independent experiments. Animal numbers per group (n) are depicted. **(F)** Weight loss of WT mice (C57BL/6) receiving doxorubicin (20mg/kg). Indicated animals were treated with 3pRNA on d-1 or were left untreated. Pooled data of 6 independent experiments. Animal numbers per group (n) are depicted. **(G)** BALB/c mice received TBI + $5x10^6$ BM cells and $15x10^6$ CFSE labeled T cells (donor C57BL/6 into recipient BALB/c). On day 4 after allo-HSCT splenic cells were analyzed by flow cytometry to identify proliferated CFSE labeled donor T cells. Shown is one representative of 2 independent experiments. Animal numbers per group (n) are depicted. **(H)** Lamina propria leukocytes (LPL) isolated from small intestines of BALB/c mice on day 8 after allo-HSCT (donor C57BL/6 into recipient BALB/c) were analyzed by flow cytometry. Indicated mice were left untreated or treated with 3pRNA on d-1. Pooled data of 3 independent experiments. Animal numbers per group (n) are depicted. **(I)** Survival of BALB/c mice that received 8.5Gy TBI + $5x10^6$ BM alone or $5x10^6$ BM and $0.5x10^6$ T cells (donor C57BL/6 into recipient BALB/c) and that were inoculated with $0.25x10^6$ A20 tumor cells. Pooled data of 2 independent experiments. Animal numbers per group (n) are depicted. **(J)** Allo-HSCT recipients were inoculated with A20-TGL and *in vivo* bioluminescence imaging was conducted to determine tumor burden. Bioluminescence of one representative experiment on d21 after allo-HSCT is shown. All experiments were analyzed using two-tailed unpaired t test or ordinary one-way Anova for multiple. Survival was analyzed using the Log-rank test. Significance was set at p values < 0.05, $p < 0.01$ and $p < 0.001$ and was then indicated with asterisks (*, ** and ***). If not otherwise indicated, significance was calculated compared to untreated groups. Data are presented as mean ± S.E.M.

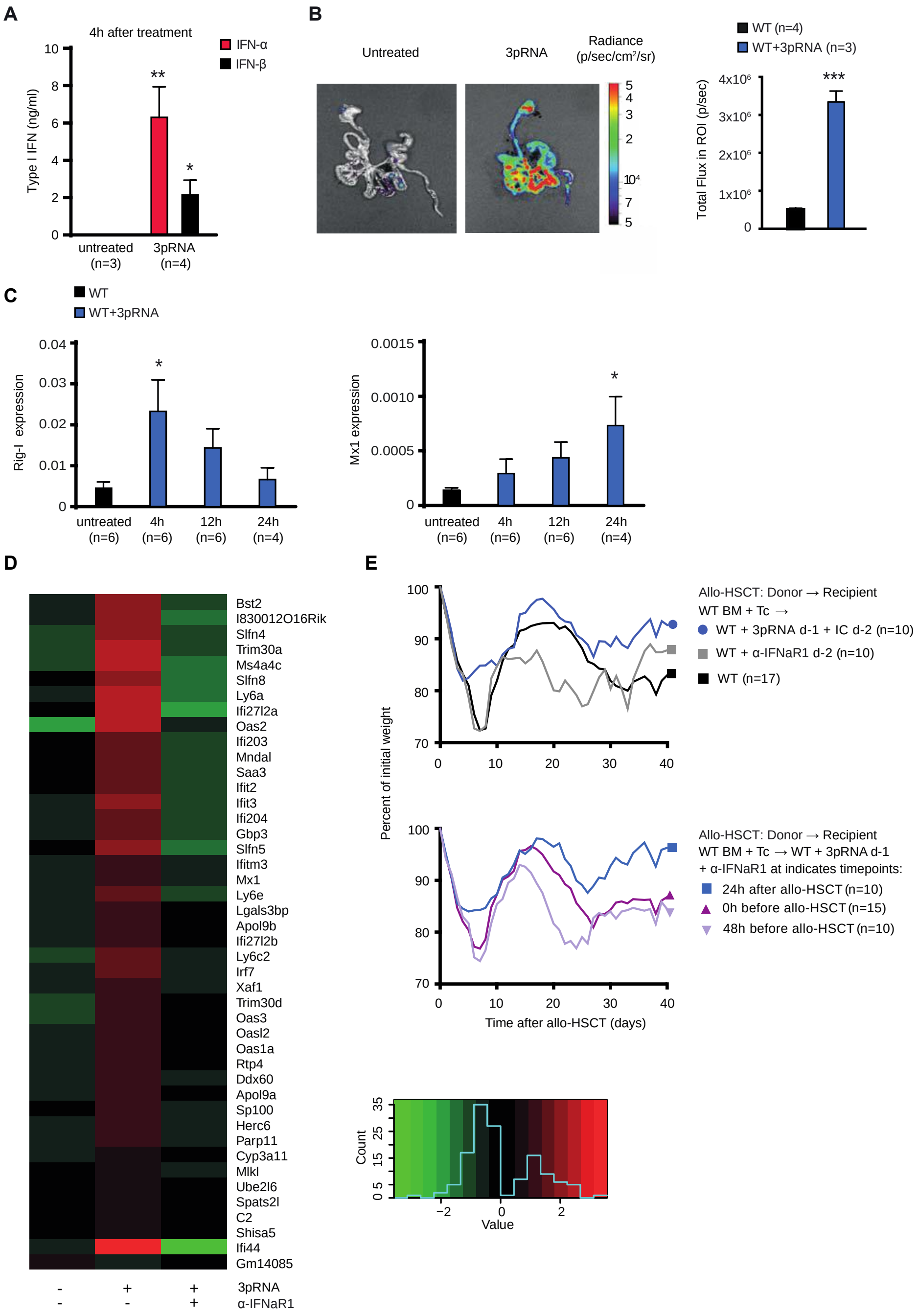
A
4h after treatment
IFN-α
IFN-β
Type I IFN (ng/ml)
untreated (n=3)
3pRNA (n=4)
B
Untreated
3pRNA
Radiance (p/sec/cm²/sr)
WT (n=4)
WT+3pRNA (n=3)
Total Flux in ROI (p/sec)
C
WT
WT+3pRNA
Rig-I expression
Mx1 expression
untreated (n=6)
4h (n=6)
12h (n=6)
24h (n=4)
D
Bst2
I830012O16Rik
Slfn4
Trim30a
Ms4a4c
Slfn8
Ly6a
Ifi27l2a
Oas2
Ifi203
Mndal
Saa3
Ifit2
Ifit3
Ifi204
Gbp3
Slfn5
Ifitm3
Mx1
Ly6e
Lgals3bp
Apol9b
Ifi27l2b
Ly6c2
Irf7
Xaf1
Trim30d
Oas3
Oasl2
Oas1a
Rtp4
Ddx60
Apol9a
Sp100
Herc6
Parp11
Cyp3a11
Mlkl
Ube2l6
Spats2l
C2
Shisa5
Ifi44
Gm14085
3pRNA
α-IFNaR1
E
Allo-HSCT: Donor → Recipient
WT BM + Tc →
WT + 3pRNA d-1 + IC d-2 (n=10)
WT + α-IFNaR1 d-2 (n=10)
WT (n=17)
Percent of initial weight
Allo-HSCT: Donor → Recipient
WT BM + Tc → WT + 3pRNA d-1
+ α-IFNaR1 at indicates timepoints:
24h after allo-HSCT (n=10)
0h before allo-HSCT (n=15)
48h before allo-HSCT (n=10)
Time after allo-HSCT (days)
Count
Value

Figure S4

Figure S4. RIG-I–induced treatment effects are mediated by IFN-Is. (**A**) Serum Type I IFN levels of untreated or 3pRNA treated BALB/c WT mice were determined 4 h after i.v. injection of 25ug 3pRNA or vehicle control (jetPEI). Animal numbers per group (n) are depicted. (**B**) Left panel: Albino C57BL/6 mice carrying an IFN-β$^{\Delta\beta\text{-luc}}$ allele were injected i.v. with 25 µg 3pRNA. 24 hours later, luciferin was injected i.v., and luciferase activity of isolated intestines was determined by bioluminescence imaging (one representative image is shown). Right Panel: The luciferase activity was quantified in a region of interest covering the small intestine (n=4 mice in untreated group; n=3 mice in the 3pRNA treated group). One representative experiment is shown. **(C)** *Rig-I* mRNA transcript expression (left panel) and *Mx1* mRNA transcript expression (right panel) was determined in small intestinal epithelial cells of untreated or 3pRNA-treated BALB/c mice at the indicated time points. Relative transcript levels were normalized to the housekeeping gene *β-Actin*. Shown are pooled data of 2 independent experiments. Animal numbers per group (n) are depicted. **(D)** Heatmap depicting interferon regulated genes of Balb/c mice that were solely irradiated (9Gy) (n=3, **left lane**), (ii) pretreated with 3pRNA prior (d-1) to irradiation (n=3, **middle lane**) or (iii) pre-treated with 3pRNA (d-1) + α-IFNaR1 blocking antibody (d-2) prior to irradiation (n=3; **right lane**). RNA from small intestines was isolated 12 h after irradiation and used for RNA sequencing. The heatmap shows all genes listed in the interferome database that show significantly changed gene expression of 3pRNA pretreated and irradiated mice compared to both the other groups simultaneously. **(E)** Weight loss of allo-HSCT recipients. Indicated mice received 3pRNA on d-1 and/or α-IFNaR1 blocking. Both upper and lower panel show pooled data of the same 3 independent experiments. The lower panel shows mice that received combination treatment of 3pRNA and α-IFNaR1 blocking Ab at indicated time points. Animal numbers per group (n) are depicted. All experiments were analyzed using two-tailed unpaired t test or ordinary one-way Anova for multiple comparisons. Survival was analyzed using the Log-rank test. Significance was set at p values < 0.05, $p < 0.01$ and $p < 0.001$ and was then indicated with asterisks (*, ** and ***). If not otherwise indicated, significance was calculated compared to untreated groups. Data are presented as mean ± S.E.M.

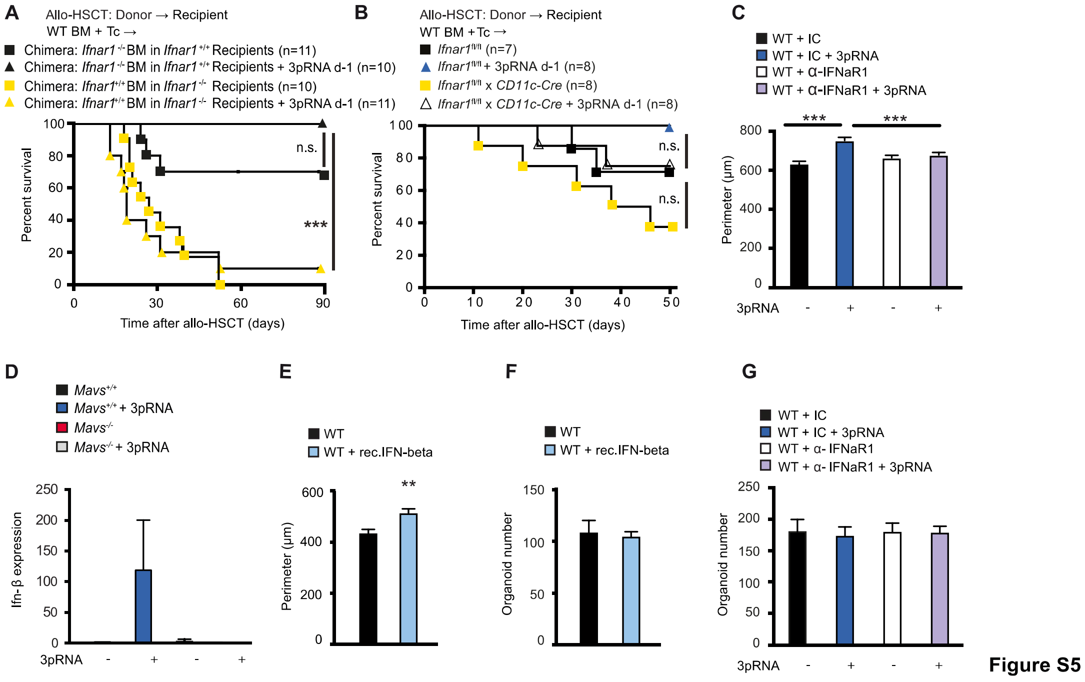

Figure S5. RIG-I–induced IFN-Is enhance epithelial regeneration through stimulation of the ISC compartment. (A) 90 days after syngeneic bonemarrow transplantation between *Ifnar1*$^{-/-}$ and *Ifnar1*$^{+/+}$ mice (C57BL/6), bonemarrow chimera were lethally irradiated and transplanted with 5×10^6 BM cells with 1×10^6T cells from donor B10.BR mice and monitored for survival. Indicated mice were either left untreated or treated with 3pRNA on d-1. Pooled data of 2 independent experiments. Animal numbers per group (n) are depicted. **(B)** Survival of either *Ifnar1*$^{fl/fl}$ or *Ifnar1*$^{fl/fl}$ *CD11cCre* allo-HSCT recipients (donor BALB/c into recipient C57BL/6) in the presence or absence of 3pRNA (d-1). Pooled data of 2 independent experiments. Animal numbers per group (n) are depicted **(C)** Measurement of organoid size (perimeter) of C57BL/6 small intestinal organoids after 5 days in culture. Indicated crypts were treated with 3pRNA (2µg/ml), α-IFNaR1 blocking Ab (10ug/ml) or IgG1 Isotype control (IC). The experiment was performed 3 times and one representative experiment is shown. **(D)** *IFN-β* mRNA transcript expression 24 hours after 3pRNA stimulation of *Mavs*$^{+/+}$ or *Mavs*$^{-/-}$ small intestinal organoids. The experiment was performed 3 times and resulting data were pooled. **(E)** Measurement of organoid size (perimeter) of C57BL/6 small intestinal organoids after 5 days in culture. Indicated crypts were treated with rec. IFN-β (20U/ml). The experiment was performed 3 times and one representative experiment is shown. (**F**) Number of organoids of C57BL/6 small intestinal organoids after 7 days in culture. Indicated Crypts were treated with rec. IFN-β (20U/ml). **(G)** Number of C57BL/6 small intestinal organoids treated as in (**C**) after 7 days in culture. Survival was analyzed using the Log-rank test. All other experiments were analyzed using two-tailed unpaired t test or ordinary one-way Anova for multiple comparisons. Significance was set at p values < 0.05, p < 0.01 and p < 0.001 and was then indicated with asterisks (*, ** and ***). Data are presented as mean ± S.E.M.

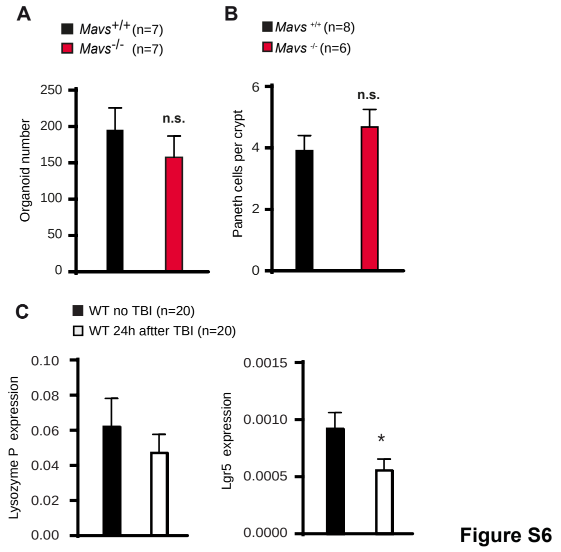

Figure S6. MAVS-deficient mice do not display an inherent defect in organoid formation or in the number of Paneth cells. (A) Number of organoids grown *ex vivo* from small intestinal crypts of *Mavs*$^{+/+}$ and *Mavs*$^{-/-}$ mice after 5 days in culture. Pooled data of 7 independent experiments. Animal numbers per group (n) are depicted. **(B)** Determination of Lysozyme^{+} paneth cells per crypt in the ileum of untreated *Mavs*$^{+/+}$ and *Mavs*$^{-/-}$ mice using immunohistochemistry (IHC). Pooled data of 2 independent experiments. Animal numbers per group (n) are depicted. **(C)** RNA of small intestines from Balb/c WT mice isolated 24 hours after irradiation (9Gy). Gene expression was determined by qPCR. Relative transcript levels of *Lysozyme* P and *Lgr5* were normalized to the housekeeping gene *β-Actin*. Pooled data of 2 independent experiments. Animal numbers per group (n) are depicted. All experiments were analyzed using two-tailed unpaired t test or ordinary one-way Anova for multiple comparisons or. Significance was set at p values < 0.05, $p < 0.01$ and $p < 0.001$ and was then indicated with asterisks (*, ** and ***). Data are presented as mean ± S.E.M.

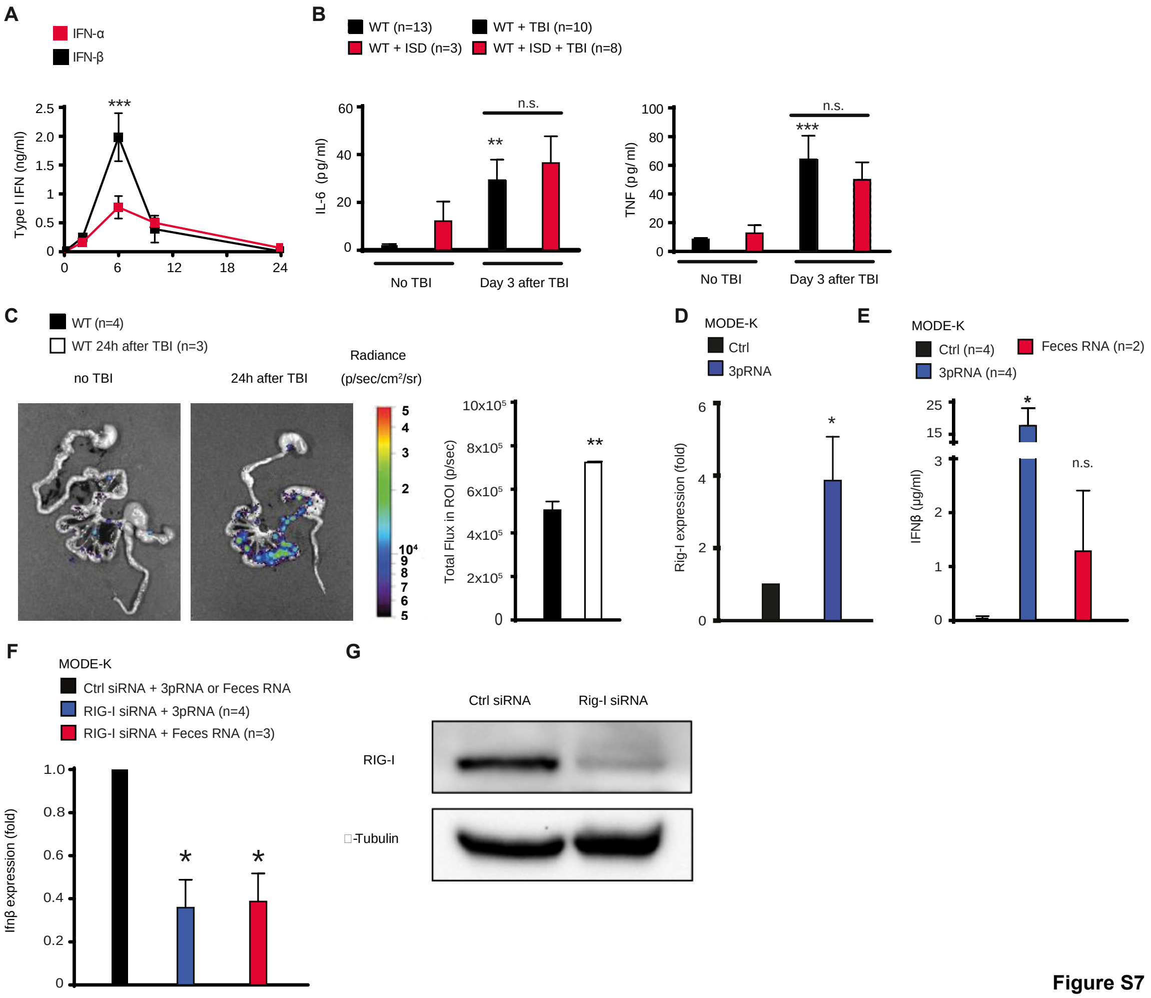

Figure S7

Figure S7. TBI and IFN-stimulatory DNA induce a systemic IFN-I response and feces-derived RNA triggers a RIG-I-dependent IFN-I response in IECs. (A) Serum levels of IFN-α and IFN-β were determined at the indicated time points (hours) after *i.v.* injection of 50µg interferon-stimulatory DNA (ISD) into C57BL/6. Pooled data of n=2 independent experiments (n=2-5 per time point). **(B)** Serum levels of IL-6 (left panel) and TNF-α (right panel) of untreated or irradiated (9Gy), ISD or TBI + ISD stimulated BALB/c mice. Serum levels of the indicated proteins were determined by cytometric bead array (CBA). Pooled data of 2 independent experiments. Animal numbers per group (n) are depicted. **(C)** Left panel: Albino C57BL/6 mice carrying an IFN-$\beta^{\Delta\beta\text{-luc}}$ allele received TBI (11Gy). 24 hours later, luciferin was injected *i.v.* and luciferase activity of isolated intestine was determined. Right Panel: The luciferase activity was quantified in a region of interest covering the small intestine (n=4 mice in untreated group; n=3 mice in the TBI group). **(D)** *Rig-I* mRNA transcript expression and (**E**) IFN-β protein expression in murine MODE-K cells 18 hours after treatment with feces-derived RNA or 3pRNA**.**

Number of pooled experiments depicted. **(F)** Ifn-β mRNA transcript expression 18 hours after stimulation of control- or *Rig-I*-siRNA transfected murine MODE-K cells with mouse feces-derived RNA or 3pRNA. Number of pooled experiments depicted. **(G)** RIG-I protein expression in control- or *Rig-I*-siRNA transfected murine MODE-K cells was analyzed using western blot. Number of pooled experiments depicted. The experiments were analyzed using two-tailed unpaired t test or ordinary one-way Anova for multiple comparisons. Significance was set at p values < 0.05, $p < 0.01$ and $p < 0.001$ and was then indicated with asterisks (*, ** and ***). Data are presented as mean ± S.E.M.
